# Amyloid-β and phosphorylated tau screening in bottlenose dolphin (*Tursiops truncatus)* and striped dolphin (*Stenella coeruleoalba)* brains from Italy reveals distinct immunohistochemical patterns correlating with age and co-morbidity

**DOI:** 10.1371/journal.pone.0314085

**Published:** 2024-11-26

**Authors:** Ksenia Orekhova, Camilla Testori, Federica Giorda, Carla Grattarola, Virginia Mattioda, Giovanni Di Guardo, Cristiano Corona, Massimo Castagnaro, Eva Sierra, Cristina Casalone, Alessandra Favole, Cinzia Centelleghe, Sandro Mazzariol

**Affiliations:** 1 Department of Comparative Biomedicine and Food Science, University of Padova, Legnaro (Padova), Italy; 2 Istituto Zooprofilattico Sperimentale del Piemonte, Liguria e Valle d’Aosta, Torino, Italy; 3 Faculty of Veterinary Medicine, University of Teramo, Località Piano d’Accio, Teramo, Italy; 4 Institute of Animal Health, University of Las Palmas de Gran Canaria, Arucas, Las Palmas, Spain; Institute of Neurophysiopathology, FRANCE

## Abstract

Cetacean brains are uniquely adapted to diving, but can be affected by diseases and exposure to toxins, triggering neurodegenerative processes that may cause stranding. Some species exhibit a significant post-reproductive lifespan (PRLS), increasing the likelihood of observing cumulative and age-related pathology. Immunohistochemistry against amyloid-β and hyperphosphorylated tau proteins is increasingly implemented to assess Alzheimer’s Disease-like neuropathology in cetaceans, but comparisons between geographically distinct populations, animals of different age groups, sex, and with concomitant pathologies are lacking. We tested 43 cetaceans’ (30 *Tursiops truncatus*; 13 *Stenella coeruleoalba*) parietal cortex, our most consistently archived cerebral tissue, in immunohistochemical analyses with amyloid-β oligomer 42 (Aβ-42) and hyperphosphorylated tau (pTau AT180 and AT8) antibodies. Aβ-42 antibody cross-reacted with plaques in three aged bottlenose and two aged striped dolphins, but was more often detected within neurons, glia, and blood vessels of all the dolphins. Histoscore comparisons between dolphins of different ages, sexes, and pathologies revealed significant correlations between older age, viral infections, and plaque presence. Protozoan cysts cross-reacted with Aβ-42 antibody. pTau signal was observed as single foci in neurons and neuropil in two young and two aged bottlenose dolphins. To our knowledge, this study is the first of its kind for the Mediterranean region and will help establish baseline understanding of physiological and pathological expression of proteins associated with human neurodegenerative disease in cetacean brains.

## 1 Introduction

Marine mammals, especially cetaceans, are often regarded as "sentinels of the sea" providing critical insights into marine ecosystem health [1]. Infectious disease, toxins, and pollution can trigger neurodegenerative mechanisms that can lead to disorientation and abnormal behaviors, sometimes resulting in strandings [2–4].

Alzheimer’s disease (AD), one of the most widespread neurodegenerative diseases (NDDs) in human beings, is characterized by the pathological aggregation of amyloid-β (Aβ) and hyperphosphorylated tau (pTau) proteins, eventually forming amyloid plaques (APs) and neurofibrillary tangles (NFT), respectively [5]. Recent studies reconsidered the idea that the presence of these proteins is solely pathogenic, demonstrating that they play crucial roles in normal conditions and only become damaging when their production or degradation is disrupted, leading to an accumulation [6, 7]. In fact, in a physiologic condition, Aβ is involved in synaptic activity and neuronal survival, while the balance of tau protein phosphorylation is essential for regulating cytoplasmic microtubules and enabling cellular growth and remodeling [8–10].

According to the most diffuse theory for the pathogenesis of AD, the accumulation of extracellular fibrillar, insoluble Aβ peptides in the brain is triggered by aging [11]. Age-dependent formation of APs, NFTs, and oligodendroglial tau has been observed in several non-human primate species [12], while non-primate animals [13], especially Carnivora species, show species-specific patterns of Aβ and pTau accumulation. Among these, aged dogs and bears exhibit the presence of APs in their brains without NFTs, while Feliformia species, such as cats, leopard cats, and cheetahs, display NFTs without AP formation, even if small granular deposits of Aβ are detected in the cerebral cortex. The concomitant accumulation of Aβ and pTau has also been observed in the brain of aged pinnipeds [5].

Recent studies have shown the presence of both APs and NFTs in the brains of cetaceans. These species, like humans, present a long post-reproductive lifespan (PRLS), which has been proposed to be more closely associated with the development of AD-like changes than chronological aging itself [14, 15]. Sacchini and colleagues [16] described APs and NFTs in three odontocete species from the Canary Islands, noting the more extensive lesions in deep-diving odontocetes (beaked whale, *Ziphius cavirostris*) and suggesting that hypoxic events may play a crucial role as risk factors for cetacean NDDs. Furthermore, the social behavior of odontocetes, characterized by highly social groups that often show caregiving support towards ill or dying pod members, can help sick or cognitively impaired animals to survive longer, allowing the pathology to progress further. Vacher and collegues [17] described concomitant AD-like lesions in three oceanic species of odontocetes (bottlenose dolphin, *Tursiops truncatus;* white-beaked dolphin, *Lagenorhynchus albirostris* and long-finned pilot whale, *Globicephala melas*) and noted that the brain areas affected were analogous to those typically affected by AD in human brains, and that the cortices were more affected than brainstem nuclei. Furthermore, the distribution of the lesions was similar to that observed in pinnipeds [5].

The rare combination of caregiving behavior and PRLS make odontocetes theoretically more likely to develop advanced stages of aging-related disorders than other wild mammals [17] and it is tempting to think of classifying cetaceans into the same NDD categories as we know from humans. Apart from age, genetic susceptibility, environmental factors, and infectious diseases can influence the development of neurodegenerative lesions [18]. Exposure to toxins and contaminants has been reported as a risk factor for AD-like pathology in cetaceans [2, 19], however, nothing is known about other contributing factors.

Further characterization of Aβ and pTau immunoreactivity in cetaceans is necessary to establish physiological baselines for each species. Monitoring and comparing geographically distinct populations, as well as investigating the potential influence of age, sex, and coexisting pathology on Aβ and pTau deposition is essential to better characterize the underlying causes and significance of NDDs in these animals.

For this study, we screened the parietal brain cortices of 30 bottlenose and 13 striped dolphins that stranded or died under human care in Italy. Immunohistochemical reactivity to Aβ-42 and pTau was tested and the dolphins compared according to species, sex, age, pathological condition, and sample age. To the best of our knowledge, this is the first overview of Aβ-42 and pTau accumulation for the Mediterranean Sea region.

## 2 Materials and methods

### 2.1 Specimens

**Fig 1:**
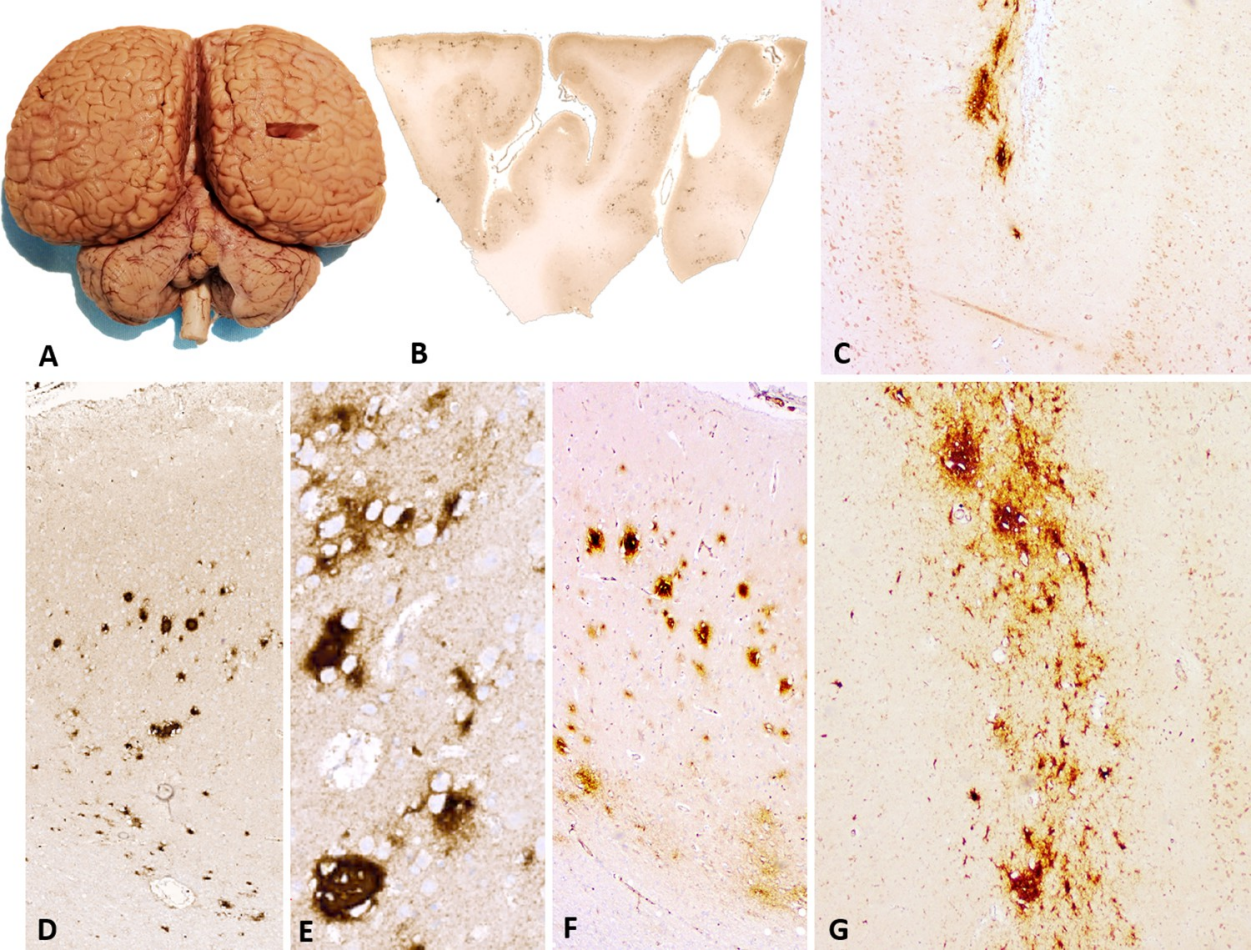
Sampling of the parietal cortex and amyloid plaques (APs). A) Dorsal view of bottlenose dolphin brain with indication of where the parietal cortex was sampled. B-G represent brain sections marked with Aβ-42 antibody: B) Overview of AP (dark brown spots) distribution of right parietal cortex section of ID 653, the bottlenose dolphin in which APs were most abundant. Magnification: 5x. C) Sulcal AP presence in layer I of striped dolphin Sc106163. Magnification: 10x. D) Presence of abundant dense-core APs across cortical layers and in the white matter of ID 653. Magnification 10x. E) APs in the telencephalon of a 9 year-old dog used as positive control tissue for the Aβ-42 antibody Magnification: 10x. F) Diffuse (top) and dense-core (bottom) AP detail in ID 653: extracellular Aβ-42 encompassed groups of 6–10 non-immunoreactive neurons. G) Clustered AP morphology in layer I Sc3908.

The dolphin brains investigated in this study came from deceased animals that had either a) stranded along the Italian shoreline (14 bottlenose and 13 striped dolphins) or b) died in facilities under human care (16 bottlenose dolphins). Only brains from dolphins with a decomposition condition code (DCC) 1 and 2 were selected from the University of Padova’s Marine Mammal Tissue Bank and from archived specimens at CReDiMa. Upon necropsy and brain extraction following the joint ACCOBAMS/ASCOBANS Best Practice guidelines [20], the largest part of the brains was placed in 10% neutral-buffered formalin for immersion fixation, while a representative subset of different brain areas (cerebrum, midbrain, cerebellum, and brainstem), were frozen for microbiological analyses. A sample of the right parietal cortex was used for subsequent analyses when available (Fig 1A). Where the right side was not available, the left side was used. No ethical approval was required for this study because tissues from deceased wildlife animals submitted for routine diagnosis were used retrospectively.

### 2.2 Immunohistochemistry

Following a morphological analysis of hematoxylin-eosin (HE) stained sections of the parietal cortex cut at 4 μm thickness, immunohistochemistry was performed using the semi-automated procedure as described by Orekhova and colleagues [21] for the Aβ-42 antibody (ab201060, abcam, Cambridge, UK). Brain tissue from aged dogs with multifocal β-amyloid plaques was used as positive control. Immunohistochemistry using pTau antibodies targeting Thr231 sites of pre-NFTs (AT180, MN1040, ThermoFisher, Renfrew, UK), and AT8 against Ser202 and Thr205 of mature NFTs (MN1020, ThermoFisher) was performed manually according to the protocol described by Vallino Costassa and colleagues [13]. Briefly, the sections were cut approximately 5 μm thick, rehydrated by routine methods and then immersed in 98% formic acid for 10 min. To enhance pTau immunoreactivity, sections were simmered in citrate buffer (pH 6.1) for 20 min. Tissues were then incubated overnight at 4°C with mouse monoclonal antibodies AT180 or AT8 (1:1000 dilution). After rinsing, a biotinylated secondary antibody (1:200 dilution; Vector Laboratories, Burlingame, CA) was applied to tissue sections for 30 min at room temperature, followed by the avidin-biotin peroxidase complex (Vectastain ABC kit; Vector Laboratories) according to the manufacturer’s protocol. Cases with APs were additionally tested with Congo Red to corroborate Aβ-42 specificity (S1 Fig). In dolphins in which protozoa-associated glial nodules, astrocytosis, or gliosis were observed in HE-stained sections, immunohistochemistry was performed using GFAP (FLEX Polyclonal Rabbit Anti-Glial Fibrillary Acidic Protein, Ready-to-Use, Dako Autostainer/Autostainer Plus; Mob 199–05, Diagnostic Biosystems, Pleasanton, CA) and Iba-1 (019–1974, Wako Chemicals USA, Richmond, VA) antibodies (S2 Fig).

In both semi-automatic and manual procedures, immunoreactivity was visualized using 3, 3’-diaminobenzidine (DakoCytomation, Carpinteria, CA) as a chromogen; sections were then counterstained with Meyer’s hematoxylin. To test the specificity of staining, primary antibodies were omitted. Each immunohistochemical run was made with an appropriate positive control. Further relevant details regarding the antibodies used are listed in Table 1.

**Table 1:** Antibodies used in this study.

Antibody	Host	Reactivity	Clonality	RRID	Catalog number	Dilution	Positive control
**Aβ-42**	Rabbit	Human, Mouse, Rat	Monoclonal	AB_2818982	ab201060	1:700	Aged dogs with multifocal APs
**Phospho-Tau (AT-180)**	Mouse	Human, Rat	Monoclonal	AB_223649	MN1040	1:1000	Human cases with AD
**Phospho-Tau (AT-8)**	Mouse	Human, Rat	Monoclonal	AB_223647	MN1020	1:1000	Human cases with AD
**GFAP**	Rabbit	Mouse, Human, Rat	Polyclonal	AB_2811722	GA524	1:500	Human case with AD
**GFAP**	Mouse	Mouse, Rabbit	Monoclonal	NA	Mob 199–05	1:20	Encephalitic dog brain
**Iba-1**	Rabbit	Human, Mouse, Rat	polyclonal	AB_839504	019–1974	1:800	Dog histiocytoma

### 2.3 Basic linear alignment of amino acid sequences

Protein sequence multiple alignments comparing human β-amyloid precursor protein (APP) [GenBank: AAB29908.1 and NCBI Reference Sequence: NP_000475.1] with APP homologs expressed by striped dolphin [GenBank: AAX81912.1], bottlenose dolphin [GenBank: AAX81917.1], and domesticated dog, *Canis lupus familiaris* [GenBank: AAX81908.1] were performed using CLUSTALW 2.1 program as previously described [22, 23]. Also, microtubule-associated protein tau sequence expressed by bottlenose dolphin [NCBI Reference Sequence: XP_033704325.1] was compared with human homolog [NCBI Reference Sequence: NP_058519.3].

### 2.4 Animal categorization and Histoscore comparisons

In the case of Aβ-42, semi-quantitative analysis could be performed based on the type and intensity score (IS) of immunoreactivity (1—mild; 2—moderate; 3—intense signal) of the parietal cortex. In the case of bottlenose dolphins, the categorization of animals based on pathological lesions (P) or absence thereof (N), as well as based on age (young adults < 30 years estimated age; old adults > 30 years-old, and calves) was consistent with that reported by Orekhova and colleagues (21). For striped dolphins, the cut-off between young and old adults was estimated to lie at 12 years old and at 195 cm of total length, based on information from Guarino and colleagues [24]. Table 2 provides an overview of the individuals considered for this study.

**Table 2:** Categorized dolphin brain specimens used in this study.

Dolphin	Stored	Species	Age	Sex	Group	Wild/human care	Pathogen	Agent	References
**ID146**	>10 years	Tt	young	M	N	H			[21]
**ID20**	>10 years	Tt	old	F	N	H			[21]
**ID89**	>10 years	Tt	young	M	N	H			[21]
**ID139**	>10 years	Tt	old	M	N	H			[21]
**ID159**	>10 years	Tt	old	M	N	H			[21]
**ID319**	>10 years	Tt	old	M	N	W			[21]
**ID344**	>5 years	Tt	young	M	N	W			[21]
**ID196**	>10 years	Tt	old	M	P	W	parasitic	*T*. *gondii*	[21]
**ID192**	>10 years	Tt	young	F	N	W			[21]
**ID107**	>10 years	Tt	young	M	P	H			[21]
**ID133**	>10 years	Tt	old	F	P	H			[21]
**ID142**	>10 years	Tt	old	F	P	W	parasitic	*T*. *gondii*	[21]
**ID165**	>10 years	Tt	old	M	P	W	parasitic	*T*. *gondii*	[21]
**ID201**	>10 years	Tt	old	M	P	W	viral	CeMV	[21, 25]
**ID203**	>10 years	Tt	old	M	N	W			[21]
**ID114**	>10 years	Tt	calves	M	calves	H			[21]
**ID144**	>10 years	Tt	calves	M	calves	H			[21]
**ID145**	>10 years	Tt	calves	M	calves	H			[21]
**ID343**	>5 years	Tt	calves	F	calves	H			[21]
**ID123**	>10 years	Tt	calves	F	calves	H			
**ID545**	<5 years	Tt	calves	M	calves	W			
**ID520**	<5 years	Tt	old	F	P	H	bacterial	*P*. *aeruginosa*	
**ID544**	<5 years	Tt	old	F	N	H			
**ID596**	<5 years	Tt	young	M	N	W			
**ID598**	<5 years	Tt	old	F	P	W	viral	Herpesvirus	
**ID624**	<5 years	Tt	young	F	N	W			
**ID653**	<5 years	Tt	old	F	P	H			
**Tt66499/21**	<5 years	Tt	young	M	P	H	bacterial	*Photobacterium damselae*	
**Tt177/22**	<5 years	Tt	old	F	P	W	viral	CeMV; (herpesvirus—not tested in brain)	[26]
**Tt51352/20**	<5 years	Tt	old	F	P	W	bacterial; parasitic	*Photobacterium damselae*; *Listeria grayi*; *Clostridium spp*.; *T*. *gondii*	[26, 27]
**Sc8319/21**	<5 years	Sc	young	M	P	W	viral	Herpesvirus	
**Sc26362/21**	<5 years	Sc	young	M	P	W	parasitic	*T*. *gondii*	
**Sc35704**	<5 years	Sc	young	M	P	W	bacterial	*B*. *ceti*	
**Sc121502**	<5 years	Sc	old	F	P	W	viral	CeMV	
**Sc11561**	<5 years	Sc	young	F	P	W	bacterial	*B*. *ceti; Aeromonas hydrophila*	[3]
**Sc95661/19**	>5 years	Sc	old	M	P	W	parasitic	*T*. *gondii*	[27]
**Sc11447**	>5 years	Sc	old	F	P	W			[4]
**Sc106163**	>5 years	Sc	old	M	P	W	viral	CeMV	
**Sc51416**	<5 years	Sc	old	F	N	W			
**Sc3908**	>5 years	Sc	old	F	P	W	viral; bacterial	CeMV; *Staphylococcus spp*.	[27]
**Sc123517**	>10 years	Sc	old	M	P	W	viral; parasitic	CeMV; *T*. *gondii*	[4, 28]
**Sc1267**	>5 years	Sc	young	F	P	W	bacterial; parasitic	*Listeria Monocytogenes*.; *B*. *ceti*; *T*. *gondii*	[27, 29, 30]
**Sc78983**	>5 years	Sc	old	F	P	W	viral; bacterial; parasitic	CeMV, *Salmonella 1,4,[5],12:i:-*., *T*. *gondii*	[4, 27, 31]

Tt = bottlenose dolphin; Sc = striped dolphin; M = male; F = female; N = no pathology detected; P = pathological; CeMV = Morbillivirus; W = wild; H = under human care

For statistical analyses of the Histoscores (H) = (1 * % of structures with IS1) + (2 * % of structures with IS2) + (3 * % of structures with IS3), two types of Aβ-42 immunoreactive structures were considered: neuronal cytoplasmic and perineuronal Aβ-42 plaques. For each animal, the 5 high-power fields (HPFs) viewed included three representative HPFs in the grey matter, and two in the white matter, as some APs were observed there. Therefore, statistical results for neuronal and plaque immunoreactivity are reported for the 5 HPF average and the 3 HPF average of the grey matter. One-way ANOVAs were used to compare parametric Histoscore averages, whereas the Kruskal-Wallis test was implemented on non-parametric Histoscore averages when three groups were being compared. Unpaired T-test (parametric) and the Wilcoxon test (non-parametric) were implemented when two groups were compared. If significant global differences (*p* values < 0.05) were detected in multiple group comparisons, Tukey HSD and Wilcoxon signed-rank tests for parametric and non-parametric data, respectively, were used to investigate which groups were different. Due to the small sample size, three levels of adjustment with increasing restriction for α-error of the Wilcoxon test were used: None, Benjamini-Hochberg and Bonferroni. Unadjusted *p* values of significant differences (α < 0.05) are reported below.

## 3 Results

Out of 43 dolphin parietal cortices tested in this screening, all were immunoreactive to Aβ-42 antibody, although the patterns of immunoreactivity differed. Three old bottlenose dolphin females, one of which had lived under human care (ID 653), and two old striped dolphins (one male and one female, respectively) were positive for Aβ-42 perineuronal plaques. Plaque morphology and distribution varied: in the bottlenose dolphin female from under human care, which was estimated to be > 59 years old, plaques were observed in both gyri and sulci across cortical layers and in the white matter (Fig 1B). Many were dense-core plaques with IS3 immunoreactivity (Fig 1D, 1E). Some plaques were diffuse (Fig 1E, top), encompassing groups of 6–10 non-reactive neurons, while cells within dense-core plaques appeared compressed (Fig 1E, bottom). While the size of the plaques was comparable, the distribution differed in the dog positive control, where denser, smaller plaques (diameter between 10–40 μm) were observed in superficial, and diffuse Aβ-42 signal in deeper cortical layers (Fig 1F). In the wild bottlenose dolphin females and in the striped dolphins, AP morphology was often diffuse to fibrillar, irregularly shaped with poorly defined borders, limited to cortical layer I, and more often observed in sulci than in gyral crowns, especially when few plaques were present (Fig 1C and 1G). In most individuals, a diffuse, light background signal (IS ≤ 1) could be detected.

**Fig 2:**
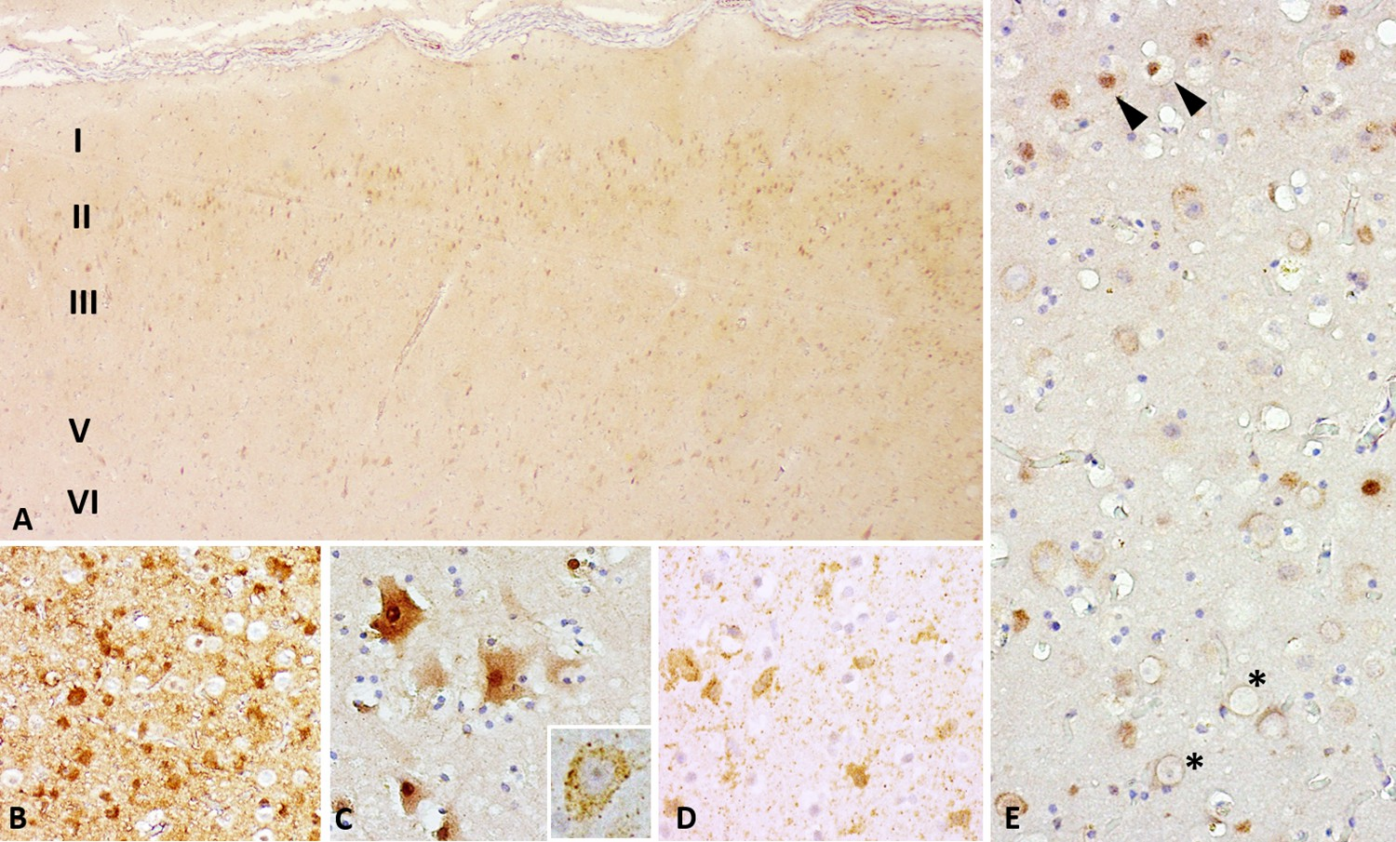
Aβ-42 immunoreactivity patterns other than APs observed in this study. A) Example of frequently observed pattern: IS1 immunoreactivity in large cortical neurons, especially in layers II, III, and V, against a light background signal (Sc8319). Multifocal vascular immunoreactivity ranging from IS0-2. Magnification: 10x. B) In some dolphins, IS2-immunoreactive neurons were present in direct proximity to non-immunoreactive neurons (Sc121502). Magnification: 20x. C) A few individuals presented both cytoplasmic and nuclear IS2-immunoreactivity, sometimes in neurons affected by satellitosis (Sc123517). Magnification: 40x. D) Multifocally, IS1-2-immunoreactive glial cells were frequently observed in the white matter (ID 598). Magnification: 40x. E) In two dolphins, nuclear signal without concurrent cytoplasmic signal (arrowheads) could be observed, in this case neighboring neurons with cytoplasmic signal of neurons with large, chromatin-poor nuclei (asterisks; Sc123517). Magnification: 40x.

However, in all dolphins apart from ID 653, cytoplasmic immunoreactivity of varying intensities (IS 0–2) was observed multifocally in the neurons, particularly visible in layers II, III, V (Fig 2A). In some cases, this signal could not be distinguished from nuclear immunoreactivity (Fig 2B), while in others, it was clearly visible in both nucleus and cytoplasm of neurons affected by satellitosis (Fig 2C). In some instances, IS2-neurons were intermixed with entirely non-immunoreactive neurons (Fig 2B). An example of an IS1-neuron is shown in the inset of Fig 2C. In two striped dolphins, nuclear signal alone was multifocally detected in some neurons (arrowheads in Fig 2E) and in the case of Sc51416, other neurons in the vicinity had conspicuously large, non-reactive neuronal nuclei (asterisk in Fig 2E). Multifocally, white matter glia (IS1–2, Fig 2D) and some blood vessels and adjacent neuropil were immunoreactive to Aβ-42, though this did not correlate with AP presence.

**Fig 3:**
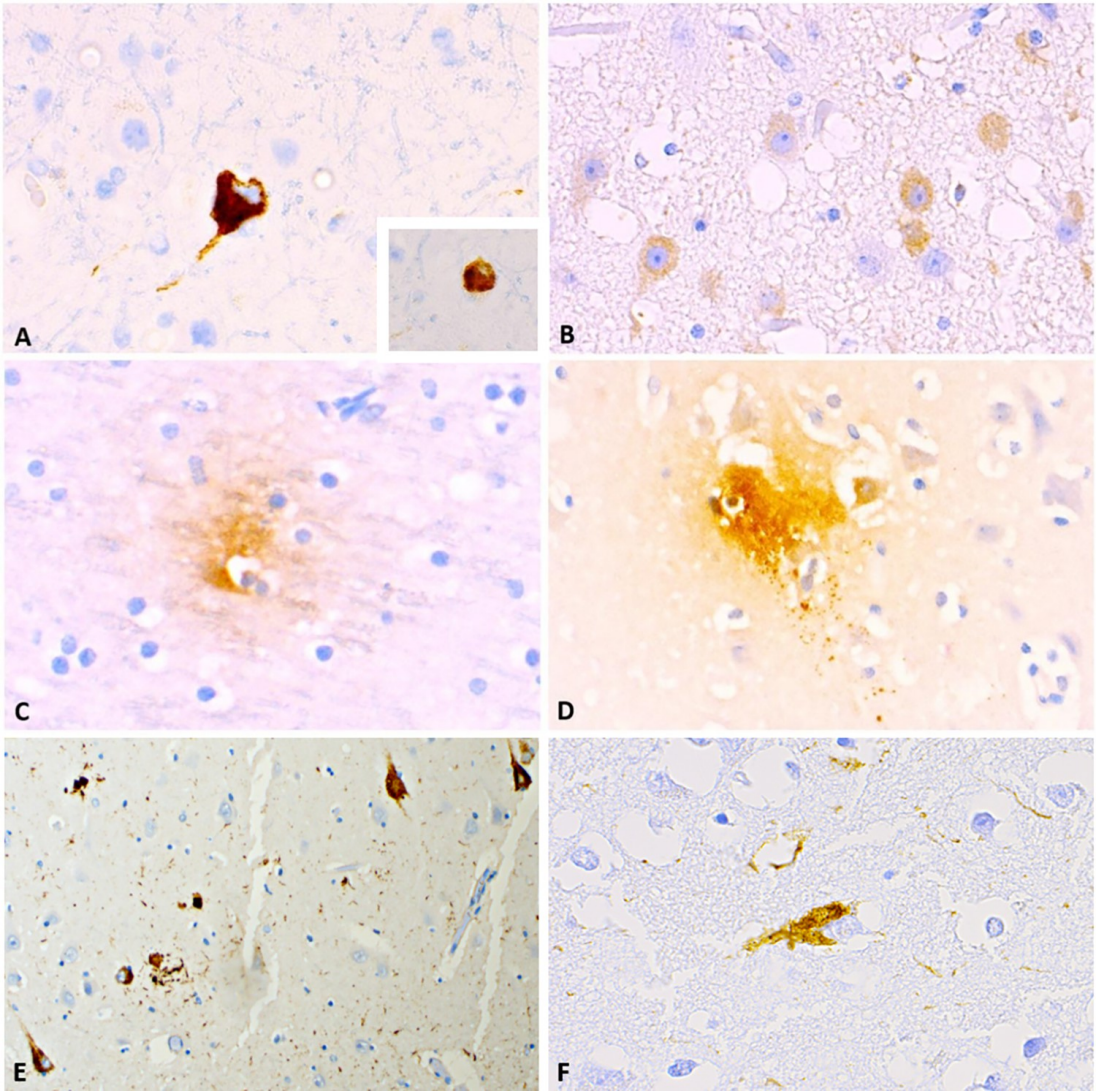
pTau immunoreactivity. A) AT8-immunoreactive NFT in single neurons and some neuropil threads in ID 653. The same area (possibly the same neuron) was positive to AT180 (inset). Magnification: 40x. B) Multifocal cytoplasmic immunoreactivity to AT180 in a young bottlenose dolphin female (ID 624). Magnification: 40x. C) Single focus of white matter immunoreactivity to AT180 in a young bottlenose dolphin male (ID 596). Magnification: 40x. D) Single focus of grey matter immunoreactivity with involvement of an adjacent neuron to AT180 in an aged bottlenose dolphin female (ID 20). Magnification: 40x. E) Multifocal cytoplasmic immunoreactivity in an AD human brain used as positive control tissue for the AT-180 antibody F) Cytoplasmic immunoreactivity in an AD human brain used as positive control tissue for the AT-8 antibody.

With regards to pTau, most investigated parietal cortices from 43 dolphins were non-immunoreactive. Only four bottlenose dolphins displayed discrete immunoreactivity to pre-NFT-associated pTau (AT180). This took the form of single, small, diffuse foci with an IS1 within grey (Fig 3D) or white matter (Fig 3C). This was visible in animals without Aβ-42 plaques, one (ID 20) being a 30-year-old female from under human care, and the other female (ID 596) a young adult from the wild. Another young adult female (ID624) displayed multifocal IS1-immunoreactive neurons (Fig 3B).

In the > 59-year-old female (ID 653), we observed single neurons with cytoplasmic signal against both AT8 and AT180 (IS1–2, Fig 3A). Comparison with AT-180 and AT-8 human positive controls are shown in Fig 3E and 3F respectively. The immunoreactivity observed was similar in distribution and intensity for AT-8, whereas for AT-180 the positive dolphin showed a weaker signal than the human control. All other dolphins were negative for pTau, therefore semi-quantitative Histoscore assessments were made only on sections immunohistochemically marked with Aβ-42-antibody, reported below.

### 3.1 Sex and age differences

**Fig 4:**
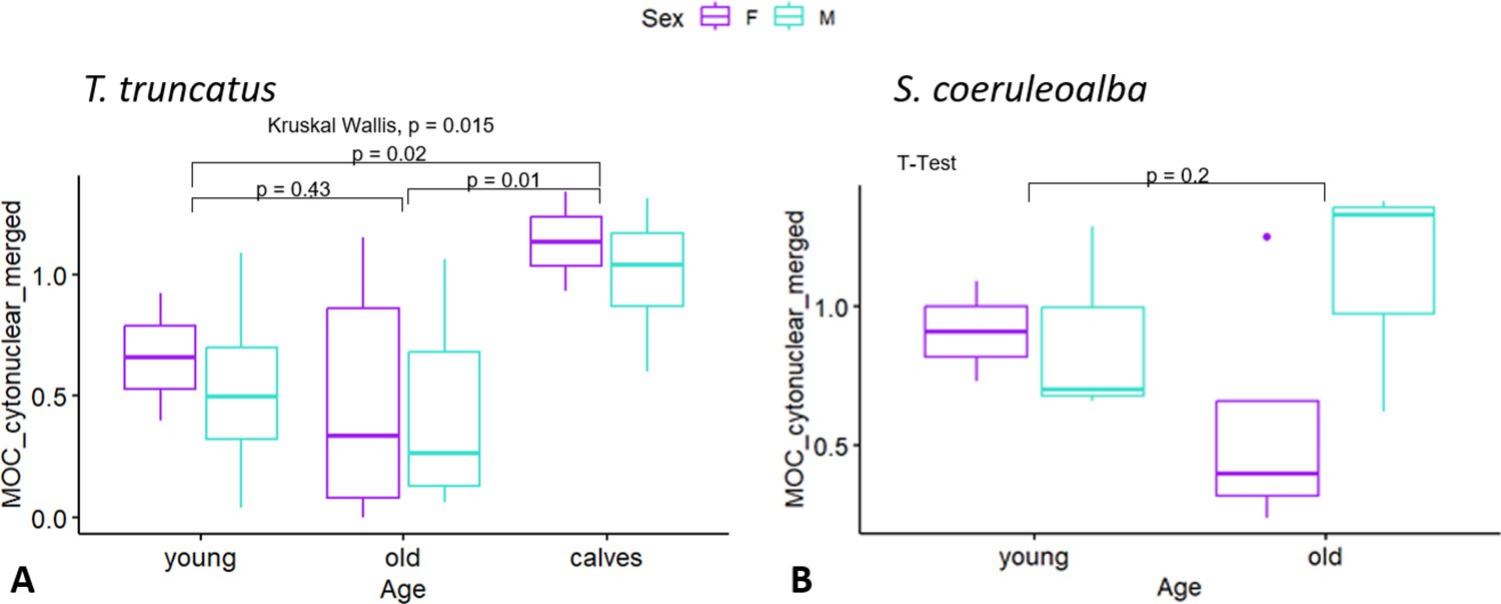
Neuronal immunoreactivity to amyloid-β relative to age and sex. Group comparisons of cytoplasmic Aβ-42 Histoscore results (y-axis) relative to the age (x-axis) and sex (color code legend above box plots) of A) bottlenose and B) striped dolphins. The box plots are visual aids to give an overview of values obtained for each age and sex group. Statistical comparisons were performed on age and sex variables separately, and sex differences were not assessed within age groups due to low sample sizes. P values displayed are those of the age comparisons.

In comparisons of neuronal cytoplasmic immunoreactivity, no significant differences owing to older age could be detected in either species (Fig 4). However, among the bottlenose dolphins, calves consistently displayed higher Histoscore averages than both young (*p = 0*.*02*) and old adults (*p = 0*.*0079*). Unadjusted *p* values are displayed in the box plots below. There were no significant sex differences.

### 3.2 Differences by pathology

**Fig 5:**
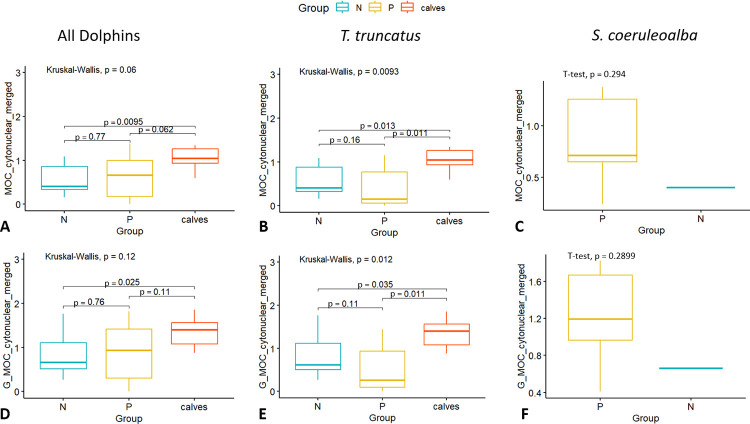
Neuronal immunoreactivity to amyloid-β relative to the presence of pathology. Group comparisons of cytoplasmic Aβ-42 Histoscore results (y-axis) relative to classification into pathological (P), non-pathological (N), and calves’ brains (x-axis) considering the total averages of 5 HPFs including white matter (A, B, C) or just grey matter (D, E, F) of both species (A, D), bottlenose (B, E), and striped dolphins (C, F).

When animals were grouped generally according to presence of (P) or no pathology (N) within the brain, the only statistically significant differences could be noted amongst both pathological and non-pathological adults and calves amongst the bottlenose dolphins, which also influenced the statistics of all dolphins taken together (Fig 5A, 5B, 5D and 5E). Calves were considered separately here, as it is unknown whether their developing brains have a different baseline from adults altogether. No calves were available for the striped dolphins, and only one non-pathological individual was included, so no meaningful comparison could be made here (Fig 5C and 5F). The next step was to assess viral, bacterial, and parasitic etiologies separately.

#### 3.2.1 Viral

**Fig 6:**
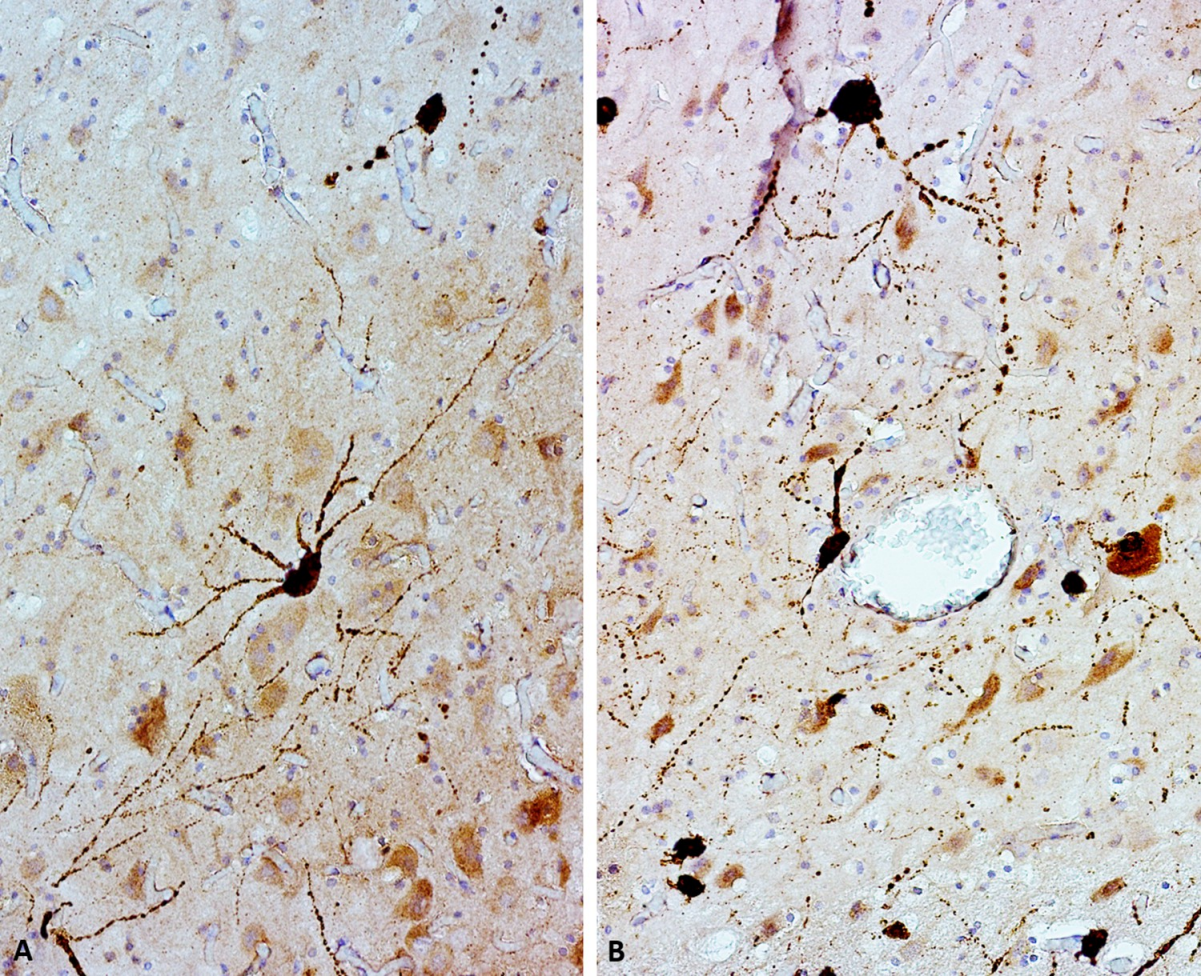
Aβ-42-immunoreactivity. Multifocally, intensely Aβ-42-immunoreactive (IS3) neurons with immunoreactivity continuing into the dendrites could be observed in a CeMV-positive bottlenose dolphin (Tt177/22). Note that here, no cross-reaction with blood cells is observed (B, figure center). Magnification: 40x.

No statistically significant differences could be detected using Histoscore comparisons for cytoplasmic immunoreactivity within neurons. However, qualitatively, one bottlenose dolphin (Tt177/22) with molecular traces of Dolphin Morbillivirus (CeMV) in its brain displayed a distinct immunoreactivity pattern against Aβ-42. Multifocally, single neurons in deeper cortical layers were intensely stained with the antibody (IS3) at the soma as well as along the dendritic processes, almost reminiscent of a “neuropil thread” as it is known to occur when using anti-pTau antibodies (Fig 6).

**Fig 7:**
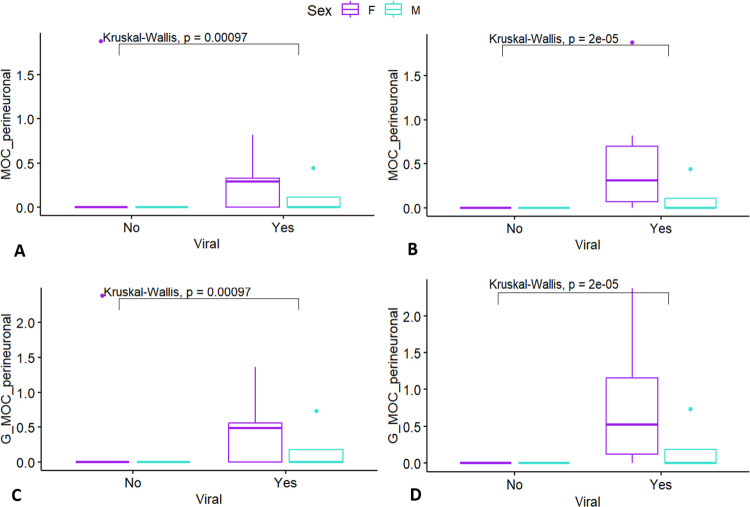
Amyloid plaques in dolphins with viral infections. Group comparisons of perineuronal Aβ-42 Histoscore results (y-axis) relative to sex (color coding) and presence of viral infections (x-axis) of the dolphins, considering the total averages of 5 HPFs including white matter (A, B) or just grey matter (C, D). In A and B, ID 653 is considered as not having viral involvement in brain pathology. In C and D, viral pathology is considered a factor in ID 653’s brain pathology. The box plots are visual aids to give an overview of values obtained for each age and sex group. Statistical comparisons were performed on age and sex variables separately, and sex differences were not assessed within age groups due to low sample sizes. P values displayed are those of the age comparisons.

Moreover, when comparing Histoscores of perineuronal plaques, a very clear pattern emerged as to the presence of viral infections (CeMV or herpesvirus) and presence of plaques in our study group, which represents a large part of the decomposition and conservation code 1–2 [20] striped and bottlenose dolphin brains sampled in Italy over the last 20 years. At first glance, all but ID 653 had viral infections detected within the brain, resulting in a significant *p* value of *p = 0*.*00097* (Fig 7A and 7C). However, this > 59-year-old female under human care had been wild-caught, and while the brain had resulted negative in PCR analyses, there had been a signal for herpesvirus in some skin lesions and, weakly, in the kidneys. ID 653 was therefore experimentally regrouped into the viral group, which resulted in a highly significant p-value (*p = 2e*^*-5*^; Fig 7B and 7D).

#### 3.2.2 Bacterial

No statistically significant group differences could be detected for bacterial presence in this investigation. This is depicted in S3 Fig (S3A and S3C Fig).

#### 3.2.3 Parasitic

No statistically significant group differences could be detected for parasitic presence in this investigation, as shown in S3 Fig (S3B and S3D Fig).

**Fig 8:**
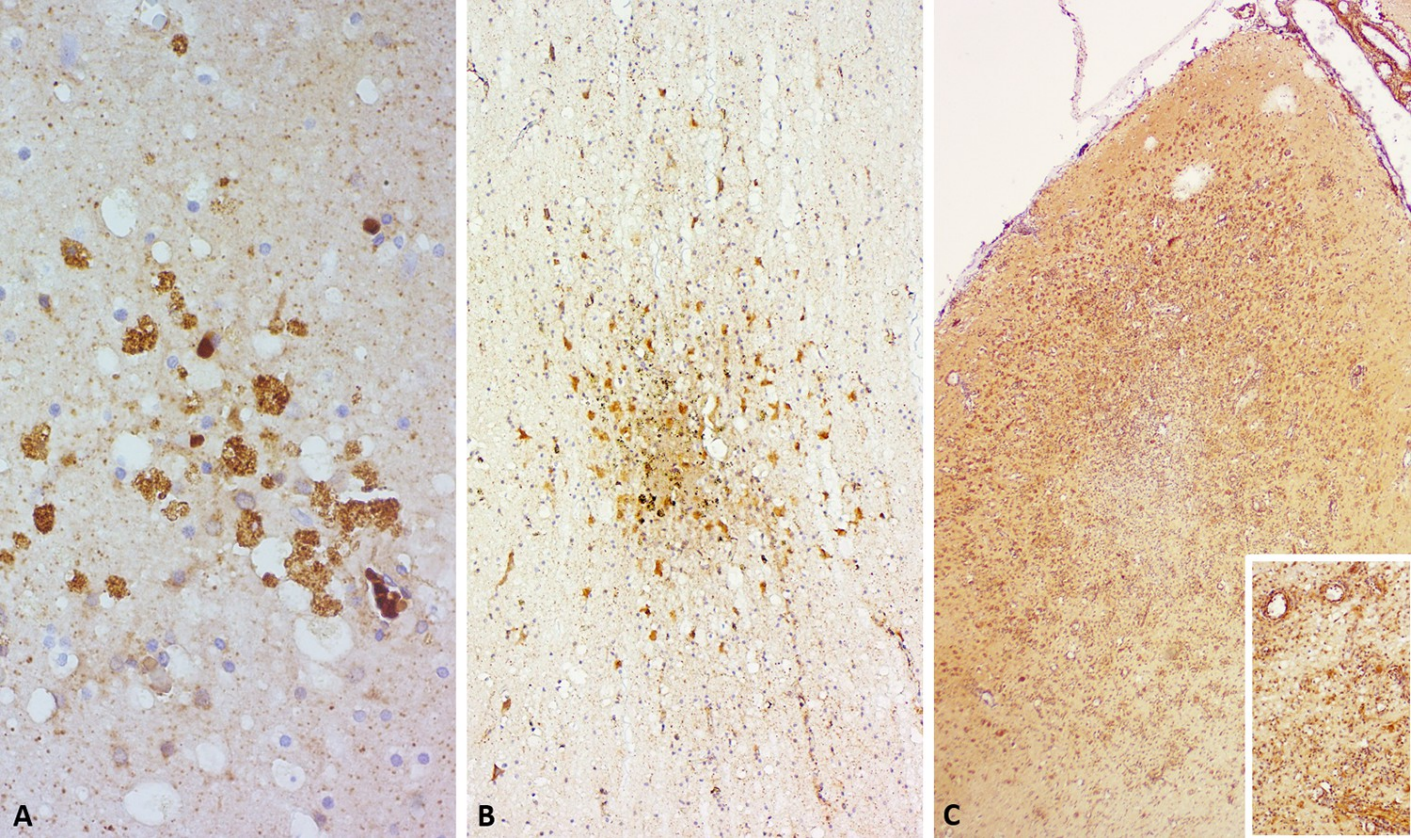
Aβ-42 immunoreactivity in *T*. *gondii*-infected dolphins. The immunoreactivity ranged from A) focal signal in microcyst-like structures, to B) foci of diffuse background signal in glial nodules with IS2-immunoreactive glial cell involvement, to C) stronger background signal and 2IS vascular (inset) and neuronal immunoreactivity in severe encephalomeningitis effacing entire gyri.

Qualitatively, however, there was frequently a cross-reaction of the Aβ-42 with microcyst-like structures in animals positive for *Toxoplasma gondii* (*T*. *gondii*) whether there were single cysts (Fig 8A), glial nodules with additionally immunoreactive, clustered glia (Fig 8B), or severe focal-extensive gyral necrosis and multifocal-coalescing lymphohistiocytic encephalitis (Fig 8C). In the most severe case (Sc95661), there was also a more intense background signal in the neuropil, many IS2-positive neurons, and endothelial as well as perivascular immunoreactivity (IS2; inset of Fig 8C).

### 3.3 Difference by sample age

**Fig 9:**
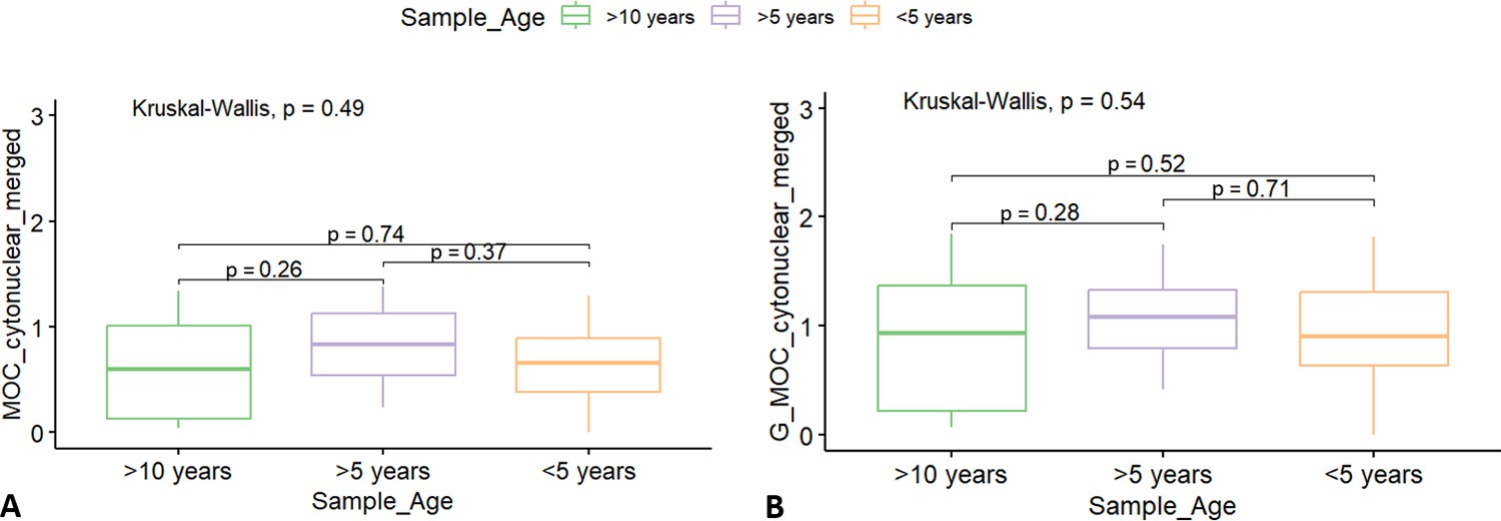
Neuronal immunoreactivity to amyloid-β relative to sample age. Group comparisons of cytoplasmic Aβ-42 Histoscore results (y-axis) in cetacean brains stored for different amounts of time after initial sampling (x-axis), considering the total averages of 5 HPFs including A) white matter or B) just grey matter in both species.

To ascertain that any perceived differences in immunoreactivity were not due to artifacts owing to lengthy storage of the samples in formalin or in paraffin blocks, pairwise comparisons were performed between tissues sampled > 10, > 5, and < 5 years before the immunohistochemical analysis. As shown in Fig 9, no significant differences could be detected.

### 3.4 Difference between dolphins from under human care and the wild

There were no significant differences between dolphins from under human care and wild dolphins (*p* values for Aβ-42 Histoscore comparisons are summarized in S2 Table).

### 3.5 Basic linear alignment of amino acid sequences

Human and dog APPs shared homology greater than 96.7% with APP expressed by striped and bottlenose dolphin, with a 100% homology between human β-amyloid from neuritic plaques of AD patients (AAB29908.1) and both dolphin and dog species. Complete sequences for microtubule-associated protein isoform 1 and X1 were only available for human and bottlenose dolphin, respectively, and shared a homology of 85.9%. The full report of CLUSTALW results is reported in S1 File.

## 4 Discussion

This multicenter study screened bottlenose and striped dolphins that died under human care or stranded along the Italian coastline for the presence and distribution of Aβ-42, pre-NFT associated pTau (AT180), and mature NFT-associated pTau (AT8) in the most consistently archived brain tissue: the parietal cortex. To ensure that any potential plaques would not be missed, a concentration higher than that of previous studies was used for Aβ-42 (1:700 as opposed to 1:25,000 reported by Vacher and colleagues [17]). While five dolphins displayed Aβ plaques, almost all tested dolphins displayed varying patterns of neuronal and glial immunoreactivity to this antibody. To the best of our knowledge, this is the first time that striped dolphins have been investigated with this combination of antibodies.

Among the 30 bottlenose dolphins examined in our study, only three old females (10% of the study group) had apparent plaques, and amongst those, the > 59-year-old female, the oldest known bottlenose dolphin under human care in Italy, had by far the most plaques. These were distributed relatively evenly across cortical layers and gyral folds, many of them with a dense-core (IS3) and some with a diffuse morphology. The plaques present in the other bottlenose dolphin females were fewer and were more likely to be found in the sulci or sides of the gyri, less so than in the gyral crowns. Meanwhile, in the striped dolphins, plaques (in two animals i.e., 15% of the study group) were distributed multifocally, but these had a more fibrillar to diffuse, clustered appearance, and when fewer were present, they would often be visible in or close to the sulci.

Preliminary studies suggest that differing plaque loads and morphologies could be linked to different apolipoprotein E genotypes [32], with *APOE ε3* alleles associated with more dense-core plaques, and *APOE ε4* alleles more frequently observed in humans with fibrillar plaques [33]. With increasing availability of sequenced cetacean genomes, future studies could elucidate whether genetic variations in these and other AD-related genes (e.g., APP, presenilins 1 and 2) [34] underlie the observed plaque morphologies.

There is some evidence that while accumulation of Aβ deposits in the human temporal lobe tend to be greater in gyral crests [35], sulcal deposits are more dense, possibly due to a higher density of neurons and blood vessels in sulcal versus gyral regions in humans [36]. In other regions of the brain like in the frontal lobe, sulci appeared to harbor more plaques than gyri [37]. To our knowledge, this neurovascular configuration has not been investigated in cetaceans. However, inquiring whether plaque clustering on a regional scale corresponds to cortical modules and specific pathways [36] could help us better understand not only the baseline functions of the dolphin brain, but how these can be impaired in neuropathological cases. In this study, it is likely that the > 59-year-old bottlenose dolphin female was the oldest dolphin examined, and the fact that the plaques were more distributed, often very dense, can be seen as compelling evidence for plaque clusters increasing and aggregating with disease progression.

It is noteworthy that all the dolphins with APs were positive for a viral infection (CeMV, herpesvirus, or both) within the brain itself (4/5 animals) or in the skin and kidneys (herpesvirus in ID 653). Considering the mode of action and tendency to latency of herpesviruses, it is likely that at some point after the infection, this virus reached the brain. Along with genetic susceptibility and environmental factors such as exposure to toxins like β-methylalanine from harmful algal blooms [2, 19], viral infections are a known risk factor for development of NDDs [34]. In *APOE ε4*-knockout mice, herpesvirus simplex (HSV1) neurotropism and latency is facilitated, and its presence within the brain is thought to induce Aβ and pTau-related pathology [38–40].

Both the striped dolphins with APs, and one bottlenose dolphin were positive to CeMV. While the exact pathogenesis of CeMV is unknown, assuming similarities to human Measles virus (MV), a persistence of viral RNA in the blood can lead to the invasion and even brain-only form of this disease in cetaceans [41, 42]. MV is associated with subacute sclerosing panencephalitis, including NFT formation, and a complex interplay of factors such as neuroinflammation, dysregulation of immune system and protein synthesis pathways is implicated in subsequent viral induction of NDDs [34, 43]. There is no evidence that the APs in the dolphins of this study are triggered by viral infection only, but considering the argument presented above, that sulci may have a high packing density of blood vessels and neurons, neuroinflammation and other metabolic disruptions induced by infectious disease could lead to a distinct pattern of AP distribution across time and depending on pathogen involved. In general, while infectious agents may trigger a pro-inflammatory state predisposing the animal to a NDD, it should be considered as one of many possible causes, and we need more specimens and brain areas sampled in cetaceans to draw clearer conclusions.

Interestingly, no significant pattern could be observed for dolphins with bacterial infections in the brain, although some authors argue that Aβ fibrillization may be an induced antimicrobial peptide-like response of the innate immune system reacting to both sterile and infectious neuroinflammatory stimuli not limited to viruses [44].

In our study, AP presence and pTau immunoreactivity showed no significant correlation. Only ID 653, the bottlenose dolphin with the most abundant APs, showed immunoreactivity to both AT180 and AT8 in consecutive sections and potentially in the same neuron, suggesting a focal presence of mature neurofibrillary tangles. However, this affected single neurons in the examined parietal cortex sections. Another aged bottlenose dolphin had an AT180-immunoreactive plaque in the grey matter, and a young bottlenose dolphin had a single small focus of immunoreactivity in the white matter, however these foci cannot be interpreted as neuritic plaques. They showed no correlation to Aβ-42-positive cells, age, or brain pathology. Different species have different combinations of Aβ-42 and pTau in older specimens’ brains–terrestrial canids often show only APs without NFTs, several pinniped species have had both APs and NFTs [5], while different brain areas studied in cetaceans have had variable degree of co-occuring APs and tauopathy. Some neuroscientific schools of thought argue that concurrent presence of Aβ in pTau-immunoreactive dystrophic neurites is necessary for the spread of AD-like neuropathology within the brain [45].

Moreover, NFTs have numerous phospho-sites, and a more comprehensive, albeit less specific, way to assess them would be to use Sevier Munger silver stain [19]. Future studies will better assess distribution and quantity immunoreactivity to these, and further, antibodies in other areas of the cetacean brain. Until then, it is too early to definitively categorize neurodegenerative phenomena in cetacean brains according to human NDD categories such as Lewy Body pathology, primary age-related tauopathy (PART), limbic‑predominant age‑related TDP‑43 encephalopathy (LATE), chronic traumatic encephalopathy (CTE), and Parkinson’s Disease [18].

Comparing our results to those of two *Grampus griseus* (Risso’s dolphin), seven G*lobicephala melas* (long-finned pilot whales), six *Lagenorhynchus albirostris* (white-beaked dolphins), five *Phocoena phocoena* (harbor porpoise) and two bottlenose dolphins from the Atlantic Ocean [17], some similarities are evident. These include: 1) the distribution of APs primarily in cortical layers I, III, and V; 2) no clear correlation between vascular Aβ-42 immunoreactivity and AP presence; 3) some dolphins without APs also being immunoreactive to AT180; 4) many neurons displayed cytoplasmic, and few intranuclear, immunoreactivity (although in our study, younger animals did not have fewer positive neurons than old dolphins–indeed the opposite was the case for bottlenose dolphin calves); 5) little glial involvement around APs was observed in both studies.

There were also notable differences: 1) the incidence of plaques per species was less variable in aged odontocetes in our study (19–25% depending on species) compared to Vacher and colleagues’ [17] analyses (20–100%) with more individuals per species considered in our study, and yet more needed to establish solid estimates of AP incidence; 2) no information on co-morbidities in the Atlantic odontocetes is reported, while in our screening, viral and parasitic infections were reflected in distinct immunoreactivity patterns using the same Aβ-42 antibody; 3) all investigated Atlantic dolphins with APs were immunoreactive to AT180, with overlapping immunohistochemical patterns–this was not the case in our study, with only single neurons with pTau signal in the dolphin with the most APs (ID 653); 4) in Vacher and colleagues [17], AT8 and AT180 immunoreactivity did not correlate, while in ID 653, these two antibodies appeared to colocalize; 5) we observed multifocal glial Aβ-42 immunoreactivity, at times aggregated in gliotic foci surrounding protozoan cysts, while this type of signal is not mentioned in the other study.

With regards to intraneuronal Aβ-42 immunoreactivity, studies on human brains reveal that this is not a reliable predictor of NDDs. Indeed, it is more often found in brain regions less susceptible to AD-like pathology, secreted by α- and β-secretases as a product of physiological cell metabolism, which is interwoven with that of APOE [10]. In people with Down syndrome and AD, neurons display reduced Aβ in neurons, thought to be the result of a shift in APP processing from in amino-terminally truncated intraneuronal Aβ to extracellular secretion of Aβ40/42 in AD patients [10]. This is reflected in two ways in our study: ID 653 exhibited abundant APs but no neuronal Aβ-42 (Fig 1F), and qualitatively, often a higher IS was observed in neurons of the gyral crests compared to sulcal neurons, which inversely correlates to the tendency of AP distribution towards the sulci or sides of the gyri, and less so in the crests.

Another reason for low or no intraneuronal Aβ (and other antigens assessed by immunohistochemistry) expression can be fixation and storage-related artifacts and loss of antigenicity. For this reason, a Histoscore comparison between brain specimens stored over many years was undertaken, and no significant differences were observed for Aβ42 in dolphin brains stored mainly as formalin-fixed, paraffin-embedded tissue for > 10, > 5, and < 5 years. Due to the insufficient number of dolphins immunoreactive against pTau, this parameter could not be assessed. In this regard, performing cetacean necropsies is often challenging due to time constraints and the location of strandings that makes difficult, if not impossible, to keep the central nervous system under ice, which would be desirable for good tissue preservation. Moreover, these challenges are compounded by varying degrees of post-mortem autolysis commonly observed in stranded cetaceans, which can affect the rate and gradient of formalin tissue penetration, as well as antigen immunoreactivity. Immunoreactivity is further influenced by the duration of tissue fixation. In our study, these factors likely contributed to the observed low pTau immunoreactivity, as well as the transient, temperature-sensitive nature of Tau phosphorylation.

Neuronal intranuclear Aβ-42 reactivity has been observed in several cetacean species [16, 17, 21], although the H31L21 Aβ antibody was shown to cross-react with a proteins corresponding to the molecular weight of APP [21], and does not show as much affinity for plaques as the mOC64 clone used in this and the study by Vacher and colleagues [17]. The significance of the intranuclear signal is not clear, however there is evidence of its effectiveness as a regulator of gene transcription [9], and some authors hypothesize a potential neuroprotective function against cellular stress such as hypoxia [7, 16]. In our study, nuclear Aβ-42 by itself was observed in two aged striped dolphins, of which one had CeMV and *T*. *gondii* infections in the brain. More often, a combination of nuclear and cytoplasmic Aβ-42 was seen, sometimes in neurons with satellitosis. We consider this to be insufficient evidence to interpret intranuclear Aβ-42 function in cetaceans, but future studies should continue noting the immunoreactivity patterns in conjunction with morphological and molecular pathology to enable a more complete comparative picture.

Moreover, as was already the case in the study of other immunohistochemical biomarkers of neuropathological lesions in cetacean brains [21], bottlenose dolphin calves have repeatedly displayed significant differences in the expression of proteins, including a higher cytoplasmic Aβ-42 Histoscore in the present study. This argues for the inclusion of various age groups and sexes in future assessments of different biomarkers generally associated with neurodegenerative processes, and the need to keep an open mind to group-specific baselines.

It is valuable to use the same antibodies and compare geographically distinct populations to refine our ability to distinguish between physiological baselines and pathological deviations of NDD-related proteins. Thereby, it is important to consider that extant cetaceans are the product of millions of years of evolution in adaptation to aquatic life and separate from that of primates, thus their baselines may deviate greatly from that of humans and other mammals. We began by looking at cetaceans as potential models for human NDDs but discovered that they likely have their own pathological patterns meriting thorough investigation. At this point in the neuroscientific exploration of cetacean brains, human neuropathological syndromes like AD, Parkinson’s Disease, PART, and others are compelling bridges in comparative neuropathology that can help to direct systematic efforts of marine mammal research.

## Supporting information

S1 Fig Congo red stain. A) Aβ-42 positive control dog and B) ID 653. Inset in (B) shows Congo Red reaction to a β-sheet structured protein around capillaries.
(TIF)

S2 Fig Glial immunoreactivity in the brains of investigated cetaceans. A) Multifocal immunoreactivity of astrocytes in glial nodules in the white matter of Toxoplasma gondii-infected striped dolphin (Sc26362) using monoclonal GFAP antibody made in mouse (Mob199-05). Magnification: 100x. B) Multifocal/coalescing astrogliosis in the grey matter of striped dolphin Sc95661 using polyclonal GFAP antibody made in rabbit. Magnification: 200x. C) Iba-1-immunoreactive microglia in ID 598 with mostly ramified morphology. Few amoeboid microglia present. Magnification: 200x.
(TIF)

S3 Fig Amyloid-β plaques in dolphins with bacterial infections. Group comparisons of perineuronal Aβ-42 Histoscore results (y-axis) relative to sex (color coding) and presence of bacterial (A, C) and parasitic (B, D) infections (x-axis) of the dolphins, considering the total averages of 5 HPFs including white matter (A, B) or just grey matter (C, D). The box plots are visual aids to give an overview of values obtained for each age and sex group. Statistical comparisons were performed on age and sex variables separately, and sex differences were not assessed within age groups due to low sample sizes. P values displayed are those of the age comparisons.
(TIF)

S1 Table Amyloid-β Histoscore results of all the cases analyzed. (XLSX)

S2 Table P-value. (XLSX)

S1 File Full report of CLUSTALW results. (DOCX)
