# Supplementary figures and images for: Amyloid-β and phosphorylated tau screening in bottlenose dolphin (Tursiops truncatus) and striped dolphin (Stenella coeruleoalba) brains from Italy reveals distinct immunohistochemical patterns correlating with age and co-morbidity
Source: PLoS One. 2024 Nov 26;19(11):e0314085. doi: 10.1371/journal.pone.0314085 (PMC11594424; doi:10.1371/journal.pone.0314085)

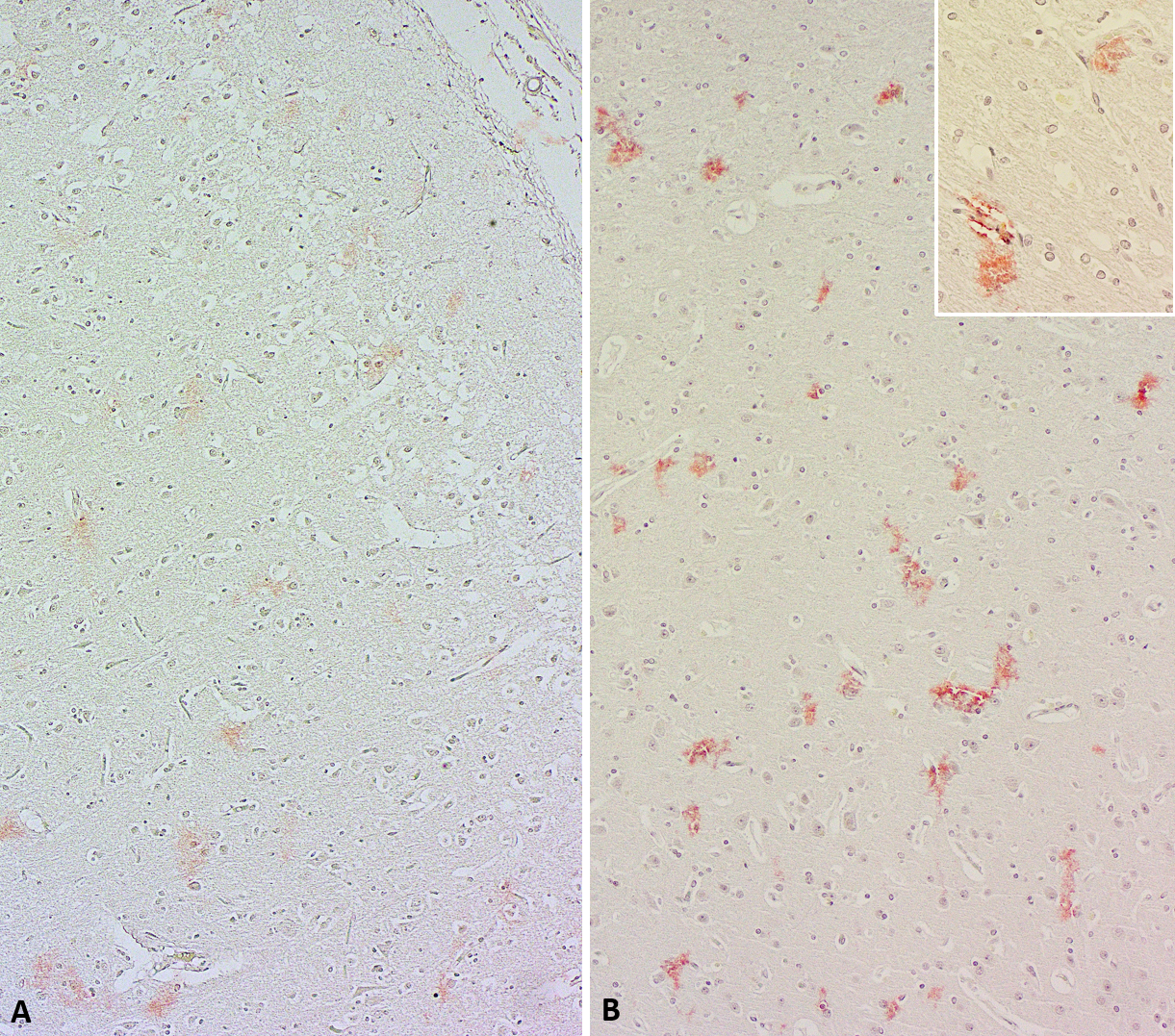

Supplement: S1 Fig — A) Aβ-42 positive control dog and B) ID 653. Inset in (B) shows Congo Red reaction to a β-sheet structured protein around capillaries. (TIF) [file pone.0314085.s001.tif]

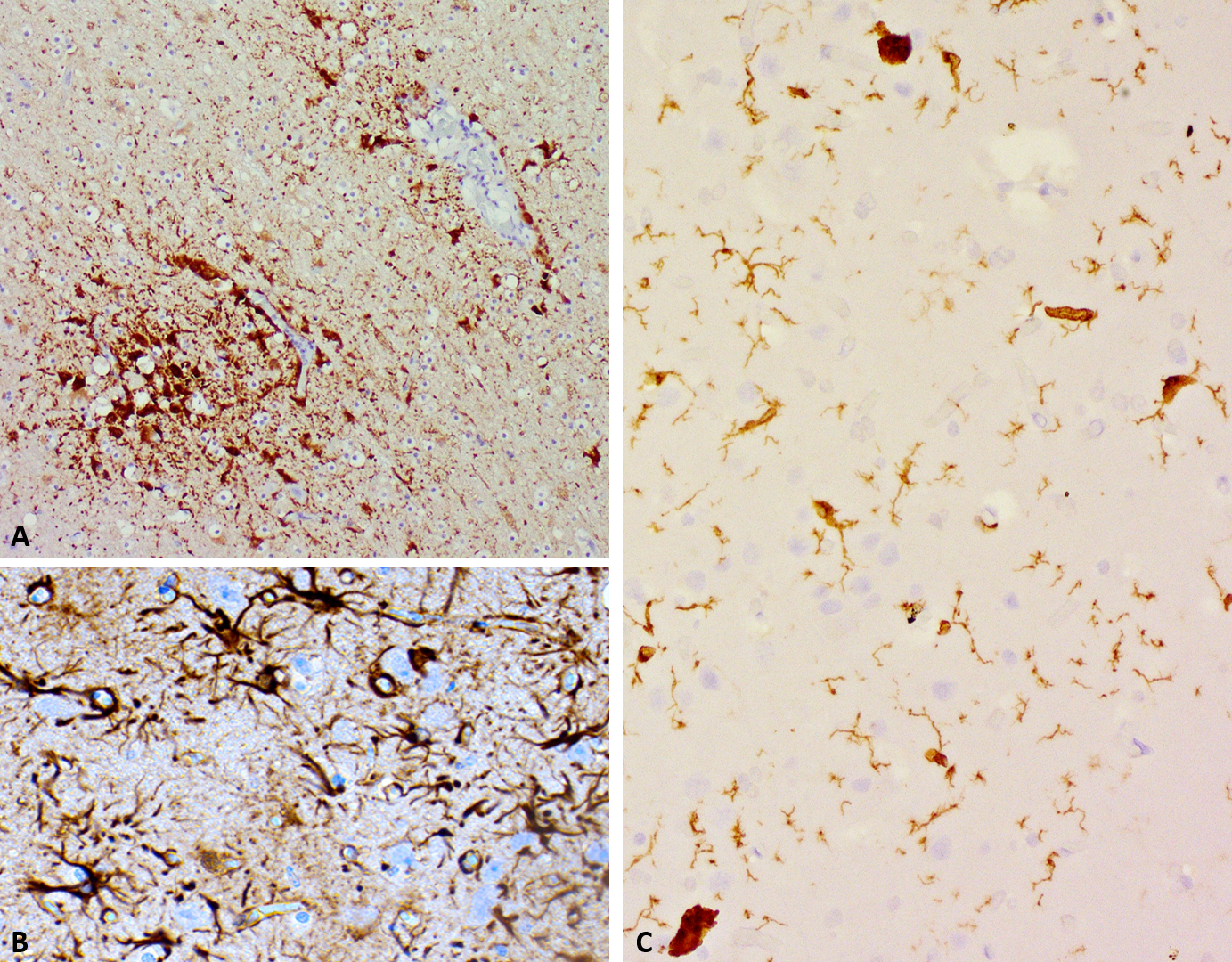

Supplement: S2 Fig — A) Multifocal immunoreactivity of astrocytes in glial nodules in the white matter of Toxoplasma gondii-infected striped dolphin (Sc26362) using monoclonal GFAP antibody made in mouse (Mob199-05). Magnification: 100x. B) Multifocal/coalescing astrogliosis in the grey matter of striped dolphin Sc95661 using polyclonal GFAP antibody made in rabbit. Magnification: 200x. C) Iba-1-immunoreactive microglia in ID 598 with mostly ramified morphology. Few amoeboid microglia present. Magnification: 200x. (TIF) [file pone.0314085.s002.tif]

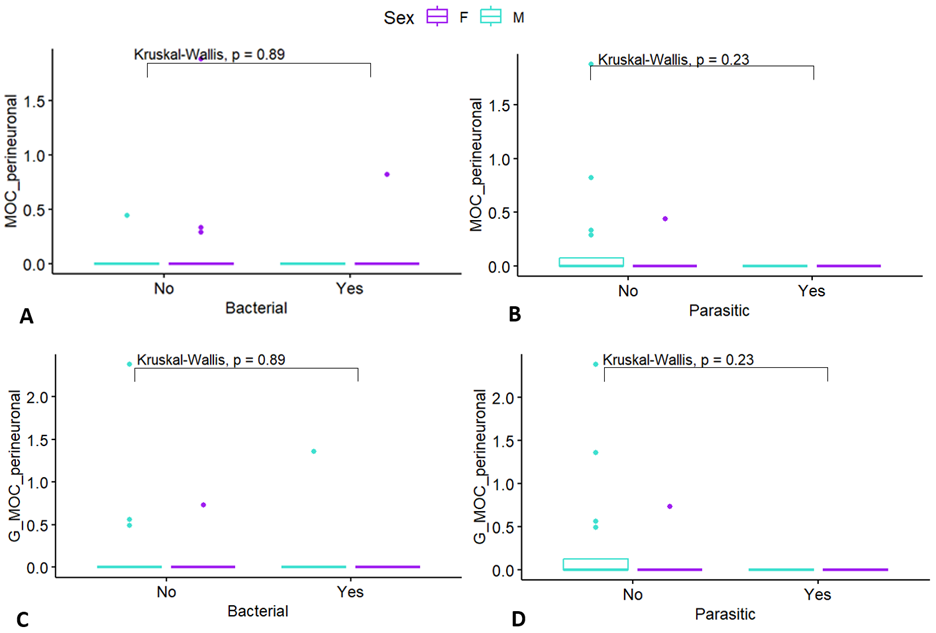

Supplement: S3 Fig — Group comparisons of perineuronal Aβ-42 Histoscore results (y-axis) relative to sex (color coding) and presence of bacterial (A, C) and parasitic (B, D) infections (x-axis) of the dolphins, considering the total averages of 5 HPFs including white matter (A, B) or just grey matter (C, D). The box plots are visual aids to give an overview of values obtained for each age and sex group. Statistical comparisons were performed on age and sex variables separately, and sex differences were not assessed within age groups due to low sample sizes. P values displayed are those of the age comparisons. (TIF) [file pone.0314085.s003.tif]
