# Supplementary material for: Amyloid-β and phosphorylated tau screening in bottlenose dolphin (Tursiops truncatus) and striped dolphin (Stenella coeruleoalba) brains from Italy reveals distinct immunohistochemical patterns correlating with age and co-morbidity
Source: PLoS One. 2024 Nov 26;19(11):e0314085. doi: 10.1371/journal.pone.0314085 (PMC11594424; doi:10.1371/journal.pone.0314085)
Supplement: S1 File — (DOCX) [file pone.0314085.s006.docx]

**beta-amyloid precursor protein 749 [Stenella coeruleoalba]**

GenBank: AAX81912.1

>AAX81912.1 beta-amyloid precursor protein 749 [Stenella coeruleoalba]

MLPGLALVLLAAWTARALEVPTDGNAGLLAEPQVAMFCGKLNMHMNVQNGKWESDPSGTKTCIGTKEGIL

QYCQEVYSELQITNVVEANQPVTIQNWCKRGRKQCKTHAHIVIPYRCLVGEFVSDALLVPDKCKFLHQER

MDVCETHLHWHTVAKETCSEKSTNLHDYGMLLPCGIDKFRGVEFVCCPLAEESDNIDSADAEEDDSDVWW

GGADTDYADGSEDKVVEVAEEEEVADVEEEEAEDEEDGDEVEEEAEEPYEEATERTTSVATTTTTTTESV

EEVVREVCSEQAETGPCRAMISRWYFDVTEGKCAPFFYGGCGGNRNNFDTEEYGMAVCGSVMSQSLLKTT

QEPLPQDPVKLPTTAASTPDAVDKYLETPGDENEHAHFQKAKERLEAKHRERMSQVMREWEEAERQAKNL

PKADKKAVIQHFQEKVESLEQEAANERQQLVETHMARVEAMLNDRRRLALENYITALQAVPPRPRHVFNM

LKKYVRAEQKDRQHTLKHFEHVRMVDPKKAAQIRSQVMTHLRVIYERMNQSLSLLYNVPAVAEEIQDEVD

ELLQKEQNYSDDVLANMISEPRISYGNDALMPSLTETKTTVELLPVNGEFSLDDLQPWHPFGVDSVPANT

ENEGSGLTNIKTEEISEVKMDAEFRHDSGYEVHHQKLVFFAEDVGSNKGAIIGLMVGGVVIATVIVITLV

MLKKKQYTSIHHGVVEVDAAVTPEERHLSKMQQNGYENPTYKFFEQMQN

**beta-amyloid precursor protein 770 [Canis lupus familiaris]**

GenBank: AAX81908.1

>AAX81908.1 beta-amyloid precursor protein 770 [Canis lupus familiaris]

MLPALALVLLASWTARALEVPTDGNAGLLAEPQVAMLCGKLRMHMNVQNGKWESDPLGTKTCIGSKEDIL

QYCQEVYPELQITNVVEANQPVTIQNWCKKGRKQCKTHAHIVIPYRCLVGEFVSDALLVPDKCKFLHQER

MDVCETHLHWHTVAKETCSEKSTNLHDYGMLLPCGIDKFRGVEFVWCPLAEESDNIDSADAEEDDSDVWW

GGADTDYADGSEDKVVEVAEEEEVADVEEEEAEDDEDDEDGDEVEEEAEEPYEEATERTTSIATTTTTTT

ESVEEVVREVCSEQAETGPCRAMISRWYFDVTEGKCAPFFYGGCGGNRNNFDTEEYCMAVCGSVMSQSLL

KTTQEPLPQDAVKLPTTAASTPDAVDKYLETPGDENEHAHFQKAKERLEAKHRERMSQVMREWEEAERQA

KNLPKADKKAVIQHFQEKVESLEQEAANERQQLVETHMARVEAMLNDRRRLALENYITALQAVPPRPRHV

FNMLKKYVRAEQKDRQHTLKHFEHVRMVDPKKAAQIRSQVMTHLRVIYERMNQSLSLLYNVPAVAEEIQD

EVDELLQKEQNYSDDVLANMISEPRISYGNDALMPSLTETKTTVELLPVNGEFSLDDLQPWHPFGVDSVP

ANTENEVEPVDARPAADRGLTTRPGSGLTNIKTEEISEVKMDAEFRHDSGYEVHHQKLVFFAEDVGSNKG

AIIGLMVGGVVIATVIVITLVMLKKKQYTSIHHGVVEVDAAVTPEERHLSKMQQNGYENPTYKFFEQMQN

**amyloid-beta precursor protein isoform a precursor [Homo sapiens]**

NCBI Reference Sequence: NP_000475.1

>NP_000475.1 amyloid-beta precursor protein isoform a precursor [Homo sapiens]

MLPGLALLLLAAWTARALEVPTDGNAGLLAEPQIAMFCGRLNMHMNVQNGKWDSDPSGTKTCIDTKEGIL

QYCQEVYPELQITNVVEANQPVTIQNWCKRGRKQCKTHPHFVIPYRCLVGEFVSDALLVPDKCKFLHQER

MDVCETHLHWHTVAKETCSEKSTNLHDYGMLLPCGIDKFRGVEFVCCPLAEESDNVDSADAEEDDSDVWW

GGADTDYADGSEDKVVEVAEEEEVAEVEEEEADDDEDDEDGDEVEEEAEEPYEEATERTTSIATTTTTTT

ESVEEVVREVCSEQAETGPCRAMISRWYFDVTEGKCAPFFYGGCGGNRNNFDTEEYCMAVCGSAMSQSLL

KTTQEPLARDPVKLPTTAASTPDAVDKYLETPGDENEHAHFQKAKERLEAKHRERMSQVMREWEEAERQA

KNLPKADKKAVIQHFQEKVESLEQEAANERQQLVETHMARVEAMLNDRRRLALENYITALQAVPPRPRHV

FNMLKKYVRAEQKDRQHTLKHFEHVRMVDPKKAAQIRSQVMTHLRVIYERMNQSLSLLYNVPAVAEEIQD

EVDELLQKEQNYSDDVLANMISEPRISYGNDALMPSLTETKTTVELLPVNGEFSLDDLQPWHSFGADSVP

ANTENEVEPVDARPAADRGLTTRPGSGLTNIKTEEISEVKMDAEFRHDSGYEVHHQKLVFFAEDVGSNKG

AIIGLMVGGVVIATVIVITLVMLKKKQYTSIHHGVVEVDAAVTPEERHLSKMQQNGYENPTYKFFEQMQN

**beta-amyloid, A beta=neuritic plaque amyloid {N-terminal} [human, familial Alzheimer's disease patient, Peptide Partial, 30 aa]**

GenBank: AAB29908.1

>AAB29908.1 beta-amyloid, A beta=neuritic plaque amyloid {N-terminal} [human, familial Alzheimer's disease patient, Peptide Partial, 30 aa]

DAEFRHDSGYEVHHQKLVFFAEDVGSNKGA

**amyloid beta protein, partial [Tursiops truncatus]**

GenBank: AAX81917.1

>AAX81917.1 amyloid beta protein, partial [Tursiops truncatus]

DAEFRHDSGYEVHHQKLVFFAEDVGSNKGAIIGLMVGGVVIA

CLUSTAL 2.1 Multiple Sequence Alignments

Sequence type explicitly set to Protein

Sequence format is Pearson

Sequence 1: AAX81908.1 770 aa

Sequence 2: NP_000475.1 770 aa

Sequence 3: AAB29908.1 30 aa

Sequence 4: AAX81917.1 42 aa

Sequence 5: AAX81912.1 749 aa

Start of Pairwise alignments

Aligning...

Sequences (1:2) Aligned. Score: 96.7532

Sequences (1:3) Aligned. Score: 100

Sequences (1:4) Aligned. Score: 100

Sequences (1:5) Aligned. Score: 97.3298

Sequences (2:3) Aligned. Score: 100

Sequences (2:4) Aligned. Score: 100

Sequences (2:5) Aligned. Score: 96.7957

Sequences (3:4) Aligned. Score: 100

Sequences (3:5) Aligned. Score: 100

Sequences (4:5) Aligned. Score: 100

Guide tree file created: [[clustalw.dnd]](https://www.genome.jp/tools-bin/pushfile?240926220045qRP7S+clustalw.dnd)

There are 4 groups

Start of Multiple Alignment

Aligning...

Group 1: Sequences: 2 Score:690

Group 2: Sequences: 3 Score:12121

Group 3: Sequences: 2 Score:503

Group 4: Sequences: 5 Score:11150

Alignment Score 15263

CLUSTAL-Alignment file created [[clustalw.aln]](https://www.genome.jp/tools-bin/pushfile?240926220045qRP7S+clustalw.aln)

[clustalw.aln](https://www.genome.jp/tools-bin/pushfile?240926220045qRP7S+clustalw.aln)

CLUSTAL 2.1 multiple sequence alignment

AAX81908.1 MLPALALVLLASWTARALEVPTDGNAGLLAEPQVAMLCGKLRMHMNVQNGKWESDPLGTK

AAX81917.1 ------------------------------------------------------------

AAX81912.1 MLPGLALVLLAAWTARALEVPTDGNAGLLAEPQVAMFCGKLNMHMNVQNGKWESDPSGTK

NP_000475.1 MLPGLALLLLAAWTARALEVPTDGNAGLLAEPQIAMFCGRLNMHMNVQNGKWDSDPSGTK

AAB29908.1 ------------------------------------------------------------

AAX81908.1 TCIGSKEDILQYCQEVYPELQITNVVEANQPVTIQNWCKKGRKQCKTHAHIVIPYRCLVG

AAX81917.1 ------------------------------------------------------------

AAX81912.1 TCIGTKEGILQYCQEVYSELQITNVVEANQPVTIQNWCKRGRKQCKTHAHIVIPYRCLVG

NP_000475.1 TCIDTKEGILQYCQEVYPELQITNVVEANQPVTIQNWCKRGRKQCKTHPHFVIPYRCLVG

AAB29908.1 ------------------------------------------------------------

AAX81908.1 EFVSDALLVPDKCKFLHQERMDVCETHLHWHTVAKETCSEKSTNLHDYGMLLPCGIDKFR

AAX81917.1 ------------------------------------------------------------

AAX81912.1 EFVSDALLVPDKCKFLHQERMDVCETHLHWHTVAKETCSEKSTNLHDYGMLLPCGIDKFR

NP_000475.1 EFVSDALLVPDKCKFLHQERMDVCETHLHWHTVAKETCSEKSTNLHDYGMLLPCGIDKFR

AAB29908.1 ------------------------------------------------------------

AAX81908.1 GVEFVWCPLAEESDNIDSADAEEDDSDVWWGGADTDYADGSEDKVVEVAEEEEVADVEEE

AAX81917.1 ------------------------------------------------------------

AAX81912.1 GVEFVCCPLAEESDNIDSADAEEDDSDVWWGGADTDYADGSEDKVVEVAEEEEVADVEEE

NP_000475.1 GVEFVCCPLAEESDNVDSADAEEDDSDVWWGGADTDYADGSEDKVVEVAEEEEVAEVEEE

AAB29908.1 ------------------------------------------------------------

AAX81908.1 EAEDDEDDEDGDEVEEEAEEPYEEATERTTSIATTTTTTTESVEEVVREVCSEQAETGPC

AAX81917.1 ------------------------------------------------------------

AAX81912.1 EAEDEE---DGDEVEEEAEEPYEEATERTTSVATTTTTTTESVEEVVREVCSEQAETGPC

NP_000475.1 EADDDEDDEDGDEVEEEAEEPYEEATERTTSIATTTTTTTESVEEVVREVCSEQAETGPC

AAB29908.1 ------------------------------------------------------------

AAX81908.1 RAMISRWYFDVTEGKCAPFFYGGCGGNRNNFDTEEYCMAVCGSVMSQSLLKTTQEPLPQD

AAX81917.1 ------------------------------------------------------------

AAX81912.1 RAMISRWYFDVTEGKCAPFFYGGCGGNRNNFDTEEYGMAVCGSVMSQSLLKTTQEPLPQD

NP_000475.1 RAMISRWYFDVTEGKCAPFFYGGCGGNRNNFDTEEYCMAVCGSAMSQSLLKTTQEPLARD

AAB29908.1 ------------------------------------------------------------

AAX81908.1 AVKLPTTAASTPDAVDKYLETPGDENEHAHFQKAKERLEAKHRERMSQVMREWEEAERQA

AAX81917.1 ------------------------------------------------------------

AAX81912.1 PVKLPTTAASTPDAVDKYLETPGDENEHAHFQKAKERLEAKHRERMSQVMREWEEAERQA

NP_000475.1 PVKLPTTAASTPDAVDKYLETPGDENEHAHFQKAKERLEAKHRERMSQVMREWEEAERQA

AAB29908.1 ------------------------------------------------------------

AAX81908.1 KNLPKADKKAVIQHFQEKVESLEQEAANERQQLVETHMARVEAMLNDRRRLALENYITAL

AAX81917.1 ------------------------------------------------------------

AAX81912.1 KNLPKADKKAVIQHFQEKVESLEQEAANERQQLVETHMARVEAMLNDRRRLALENYITAL

NP_000475.1 KNLPKADKKAVIQHFQEKVESLEQEAANERQQLVETHMARVEAMLNDRRRLALENYITAL

AAB29908.1 ------------------------------------------------------------

AAX81908.1 QAVPPRPRHVFNMLKKYVRAEQKDRQHTLKHFEHVRMVDPKKAAQIRSQVMTHLRVIYER

AAX81917.1 ------------------------------------------------------------

AAX81912.1 QAVPPRPRHVFNMLKKYVRAEQKDRQHTLKHFEHVRMVDPKKAAQIRSQVMTHLRVIYER

NP_000475.1 QAVPPRPRHVFNMLKKYVRAEQKDRQHTLKHFEHVRMVDPKKAAQIRSQVMTHLRVIYER

AAB29908.1 ------------------------------------------------------------

AAX81908.1 MNQSLSLLYNVPAVAEEIQDEVDELLQKEQNYSDDVLANMISEPRISYGNDALMPSLTET

AAX81917.1 ------------------------------------------------------------

AAX81912.1 MNQSLSLLYNVPAVAEEIQDEVDELLQKEQNYSDDVLANMISEPRISYGNDALMPSLTET

NP_000475.1 MNQSLSLLYNVPAVAEEIQDEVDELLQKEQNYSDDVLANMISEPRISYGNDALMPSLTET

AAB29908.1 ------------------------------------------------------------

AAX81908.1 KTTVELLPVNGEFSLDDLQPWHPFGVDSVPANTENEVEPVDARPAADRGLTTRPGSGLTN

AAX81917.1 ------------------------------------------------------------

AAX81912.1 KTTVELLPVNGEFSLDDLQPWHPFGVDSVPANTE------------------NEGSGLTN

NP_000475.1 KTTVELLPVNGEFSLDDLQPWHSFGADSVPANTENEVEPVDARPAADRGLTTRPGSGLTN

AAB29908.1 ------------------------------------------------------------

AAX81908.1 IKTEEISEVKMDAEFRHDSGYEVHHQKLVFFAEDVGSNKGAIIGLMVGGVVIATVIVITL

AAX81917.1 -----------DAEFRHDSGYEVHHQKLVFFAEDVGSNKGAIIGLMVGGVVIA-------

AAX81912.1 IKTEEISEVKMDAEFRHDSGYEVHHQKLVFFAEDVGSNKGAIIGLMVGGVVIATVIVITL

NP_000475.1 IKTEEISEVKMDAEFRHDSGYEVHHQKLVFFAEDVGSNKGAIIGLMVGGVVIATVIVITL

AAB29908.1 -----------DAEFRHDSGYEVHHQKLVFFAEDVGSNKGA-------------------

******************************

AAX81908.1 VMLKKKQYTSIHHGVVEVDAAVTPEERHLSKMQQNGYENPTYKFFEQMQN

AAX81917.1 --------------------------------------------------

AAX81912.1 VMLKKKQYTSIHHGVVEVDAAVTPEERHLSKMQQNGYENPTYKFFEQMQN

NP_000475.1 VMLKKKQYTSIHHGVVEVDAAVTPEERHLSKMQQNGYENPTYKFFEQMQN

AAB29908.1 --------------------------------------------------

[clustalw.dnd](https://www.genome.jp/tools-bin/pushfile?240926220045qRP7S+clustalw.dnd)

(

(

AAX81908.1:0.01073,

AAX81917.1:-0.01073)

:0.00272,

(

NP_000475.1:0.01075,

AAB29908.1:-0.01075)

:0.00539,

AAX81912.1:0.01063);

**microtubule-associated protein tau isoform X1 [Tursiops truncatus]**

NCBI Reference Sequence: XP_033704325.1

>XP_033704325.1 microtubule-associated protein tau isoform X1 [Tursiops truncatus]

MAEPRQEFNVMEDHAQGDYTLLQDHEGDVEHGLKESPLQTPADDGSEEPGSETSDAKSTPTAEDVTAPLV

DEGAPGEQAAAQPPTEIPEGTTAEEAGIGDTPNLEDQAAGHVTQARMVSKGKDGAGPEDKKTKTSTPSSA

KTLSHRPCLSSKRPTPGSSDPLIKPSSPAVCPEPSSSPKHASSVTPRTGSSGAKEMKVKGADGKPGTKIA

TPRGAAPPGQKGPANATRIPAKTTPTQKTTPSPKTPPGTGESGKSGDRSGYSSPGSPGTPGSRSRTPSLP

TPPTREPKKVAVVRTPPKSPSAAKSRLQTAPGPMPDLKNVKSKIGSTENLKHQPGGGKVQIINKKLDLSN

VQSKCGSKDNIKHVPGGGSVQIVYKPVDLSKVTSKCGSLGNIHHKPGGGQVEVKSEKLDFKERVQSKIGS

LDNITHVPGGGHKKIETHKLTFRENAKAKTDHGAEIVYKSPVVSGDTSPRHLSNVSSTGSIDMVDSPQLA

TLADEVSASLAKQGL

**microtubule-associated protein tau isoform 1 [Homo sapiens]**

NCBI Reference Sequence: NP_058519.3

>NP_058519.3 microtubule-associated protein tau isoform 1 [Homo sapiens]

MAEPRQEFEVMEDHAGTYGLGDRKDQGGYTMHQDQEGDTDAGLKESPLQTPTEDGSEEPGSETSDAKSTP

TAEDVTAPLVDEGAPGKQAAAQPHTEIPEGTTAEEAGIGDTPSLEDEAAGHVTQEPESGKVVQEGFLREP

GPPGLSHQLMSGMPGAPLLPEGPREATRQPSGTGPEDTEGGRHAPELLKHQLLGDLHQEGPPLKGAGGKE

RPGSKEEVDEDRDVDESSPQDSPPSKASPAQDGRPPQTAAREATSIPGFPAEGAIPLPVDFLSKVSTEIP

ASEPDGPSVGRAKGQDAPLEFTFHVEITPNVQKEQAHSEEHLGRAAFPGAPGEGPEARGPSLGEDTKEAD

LPEPSEKQPAAAPRGKPVSRVPQLKARMVSKSKDGTGSDDKKAKTSTRSSAKTLKNRPCLSPKHPTPGSS

DPLIQPSSPAVCPEPPSSPKYVSSVTSRTGSSGAKEMKLKGADGKTKIATPRGAAPPGQKGQANATRIPA

KTPPAPKTPPSSGEPPKSGDRSGYSSPGSPGTPGSRSRTPSLPTPPTREPKKVAVVRTPPKSPSSAKSRL

QTAPVPMPDLKNVKSKIGSTENLKHQPGGGKVQIINKKLDLSNVQSKCGSKDNIKHVPGGGSVQIVYKPV

DLSKVTSKCGSLGNIHHKPGGGQVEVKSEKLDFKDRVQSKIGSLDNITHVPGGGNKKIETHKLTFRENAK

AKTDHGAEIVYKSPVVSGDTSPRHLSNVSSTGSIDMVDSPQLATLADEVSASLAKQGL

CLUSTAL 2.1 Multiple Sequence Alignments

Sequence type explicitly set to Protein

Sequence format is Pearson

Sequence 1: XP_033704325.1 505 aa

Sequence 2: NP_058519.3 758 aa

Start of Pairwise alignments

Aligning...

Sequences (1:2) Aligned. Score: 85.9406

Guide tree file created: [[clustalw.dnd]](https://www.genome.jp/tools-bin/pushfile?241005212206puJPx+clustalw.dnd)

There are 1 groups

Start of Multiple Alignment

Aligning...

Group 1: Sequences: 2 Score:7512

Alignment Score 2703

CLUSTAL-Alignment file created [[clustalw.aln]](https://www.genome.jp/tools-bin/pushfile?241005212206puJPx+clustalw.aln)

[clustalw.aln](https://www.genome.jp/tools-bin/pushfile?241005212206puJPx+clustalw.aln)

CLUSTAL 2.1 multiple sequence alignment

XP_033704325.1 MAEPRQEFNVMEDHA----------QGDYTLLQDHEGDVEHGLKESPLQTPADDGSEEPG

NP_058519.3 MAEPRQEFEVMEDHAGTYGLGDRKDQGGYTMHQDQEGDTDAGLKESPLQTPTEDGSEEPG

********:****** **.**: **:***.: **********::*******

XP_033704325.1 SETSDAKSTPTAEDVTAPLVDEGAPGEQAAAQPPTEIPEGTTAEEAGIGDTPNLEDQAAG

NP_058519.3 SETSDAKSTPTAEDVTAPLVDEGAPGKQAAAQPHTEIPEGTTAEEAGIGDTPSLEDEAAG

**************************:****** ******************.***:***

XP_033704325.1 HVT---------------------------------------------------------

NP_058519.3 HVTQEPESGKVVQEGFLREPGPPGLSHQLMSGMPGAPLLPEGPREATRQPSGTGPEDTEG

***

XP_033704325.1 ------------------------------------------------------------

NP_058519.3 GRHAPELLKHQLLGDLHQEGPPLKGAGGKERPGSKEEVDEDRDVDESSPQDSPPSKASPA

XP_033704325.1 ------------------------------------------------------------

NP_058519.3 QDGRPPQTAAREATSIPGFPAEGAIPLPVDFLSKVSTEIPASEPDGPSVGRAKGQDAPLE

XP_033704325.1 ------------------------------------------------------------

NP_058519.3 FTFHVEITPNVQKEQAHSEEHLGRAAFPGAPGEGPEARGPSLGEDTKEADLPEPSEKQPA

XP_033704325.1 --------------QARMVSKGKDGAGPEDKKTKTSTPSSAKTLSHRPCLSSKRPTPGSS

NP_058519.3 AAPRGKPVSRVPQLKARMVSKSKDGTGSDDKKAKTSTRSSAKTLKNRPCLSPKHPTPGSS

:******.***:*.:***:**** ******.:*****.*:******

XP_033704325.1 DPLIKPSSPAVCPEPSSSPKHASSVTPRTGSSGAKEMKVKGADGKPGTKIATPRGAAPPG

NP_058519.3 DPLIQPSSPAVCPEPPSSPKYVSSVTSRTGSSGAKEMKLKGADG--KTKIATPRGAAPPG

****:**********.****:.****.***********:***** *************

XP_033704325.1 QKGPANATRIPAKTTPTQKTTPSPKTPPGTGESGKSGDRSGYSSPGSPGTPGSRSRTPSL

NP_058519.3 QKGQANATRIPAKTP------PAPKTPPSSGEPPKSGDRSGYSSPGSPGTPGSRSRTPSL

*** **********. *:*****.:**. **************************

XP_033704325.1 PTPPTREPKKVAVVRTPPKSPSAAKSRLQTAPGPMPDLKNVKSKIGSTENLKHQPGGGKV

NP_058519.3 PTPPTREPKKVAVVRTPPKSPSSAKSRLQTAPVPMPDLKNVKSKIGSTENLKHQPGGGKV

**********************:********* ***************************

XP_033704325.1 QIINKKLDLSNVQSKCGSKDNIKHVPGGGSVQIVYKPVDLSKVTSKCGSLGNIHHKPGGG

NP_058519.3 QIINKKLDLSNVQSKCGSKDNIKHVPGGGSVQIVYKPVDLSKVTSKCGSLGNIHHKPGGG

************************************************************

XP_033704325.1 QVEVKSEKLDFKERVQSKIGSLDNITHVPGGGHKKIETHKLTFRENAKAKTDHGAEIVYK

NP_058519.3 QVEVKSEKLDFKDRVQSKIGSLDNITHVPGGGNKKIETHKLTFRENAKAKTDHGAEIVYK

************:*******************:***************************

XP_033704325.1 SPVVSGDTSPRHLSNVSSTGSIDMVDSPQLATLADEVSASLAKQGL

NP_058519.3 SPVVSGDTSPRHLSNVSSTGSIDMVDSPQLATLADEVSASLAKQGL

**********************************************

[clustalw.dnd](https://www.genome.jp/tools-bin/pushfile?241005212206puJPx+clustalw.dnd)

(XP_033704325.1:0.070297,NP_058519.3:0.070297);
